# Predictive Algal Systems Biology: Integrating Omics, Genome-Scale Metabolic Models, and Machine Learning

**DOI:** 10.3390/bioengineering13060633

**Published:** 2026-05-28

**Authors:** Diego Tec-Campos, Manish Kumar, Natalia Parra, Alan Bracamonte, Ana Castillo-Sanchez, Junsu Pae, Cristal Zuñiga, Karsten Zengler

**Affiliations:** 1 Department of Pediatrics, University of California, 9500 Gilman Drive, San Diego, CA 92093, USA; 2 Faculty of Chemical Engineering, Universidad Autónoma de Yucatán, Mérida 97203, Mexico; 3 Department of Biology, San Diego State University, 5500 Campanile Dr, San Diego, CA 92182, USA; 4 DOE Great Lakes Bioenergy Research Center, San Diego State University, 5500 Campanile Dr, San Diego, CA 92182, USA; 5 Department of Bioengineering, University of California, 9500 Gilman Drive, San Diego, CA 92093, USA; 6 Center for Microbiome Innovation, University of California, 9500 Gilman Drive, San Diego, CA 92093, USA

**Keywords:** systems biology, genome-scale models, algal systems, data-driven modeling, omics tools

## Abstract

Algae represent one of the most metabolically diverse and ecologically significant groups of photosynthetic organisms, contributing fundamentally to global biogeochemical cycles while offering major potential for biotechnology applications such as biofuels, nutraceuticals, wastewater remediation, and carbon capture. However, the complexity of algal metabolism, driven by evolutionary diversity, compartmentalized cellular organization, and strong environmental coupling, makes predictive understanding of their physiology challenging. In recent years, systems biology approaches combining omics technologies, genome-scale metabolic models, and data-driven methods have begun to transform algal research from descriptive studies toward predictive frameworks. This review summarizes the current state of algal systems biology, highlighting advances in genomics, transcriptomics, proteomics, and metabolomics that enable mechanistic insights into metabolic regulation and environmental adaptation. We discuss the development, curation, and application of algal GEMs across diverse lineages, emphasizing their role in predicting metabolic flux distributions, nutrient utilization, and lipid biosynthesis. In parallel, machine learning and artificial intelligence approaches have emerged to model algal growth and cultivation performance from large physiological datasets. Finally, we discuss emerging hybrid modeling strategies that integrate mechanistic metabolic networks with data-driven predictions, outlining how these frameworks can enable next-generation predictive algal biotechnology and guide rational design of cultivation and metabolic engineering strategies.

## 1. The Dual Role of Algae in Shaping Ecosystems and Enabling Industrial Biotechnology

For over two billion years, algae have shaped the biochemistry and life of our planet as the architects of oxygenic photosynthesis and the foundation of aquatic food webs. Originating from multiple endosymbiotic events that gave rise to chloroplasts, algal lineages diversified into an extraordinary spectrum of organisms that now inhabit virtually every illuminated environment on Earth, from open oceans and freshwater lakes to desert crusts and glacial ice [1,2]. This diversification produced distinct evolutionary clades such as green algae (Chlorophyta); red algae (Rhodophyta), adapted to absorb primarily green wavelengths through phycoerythrin, while also utilizing blue light via chlorophyll a, enabling efficient photosynthesis in deeper marine environments where blue-green light predominates; brown algae and diatoms (Heterokontophyta), which combine unique chlorophyll–carotenoid pigment complexes with silica-based cell walls to dominate coastal and pelagic ecosystems; and smaller lineages such as dinoflagellates, cryptophytes, and haptophytes, which fill specialized ecological niches [3,4]. Collectively, these organisms account for nearly half of the global primary production and drive major biogeochemical cycles that regulate the planet’s climate, carbon sequestration, and nutrient turnover [1,5].

**Figure 1:**
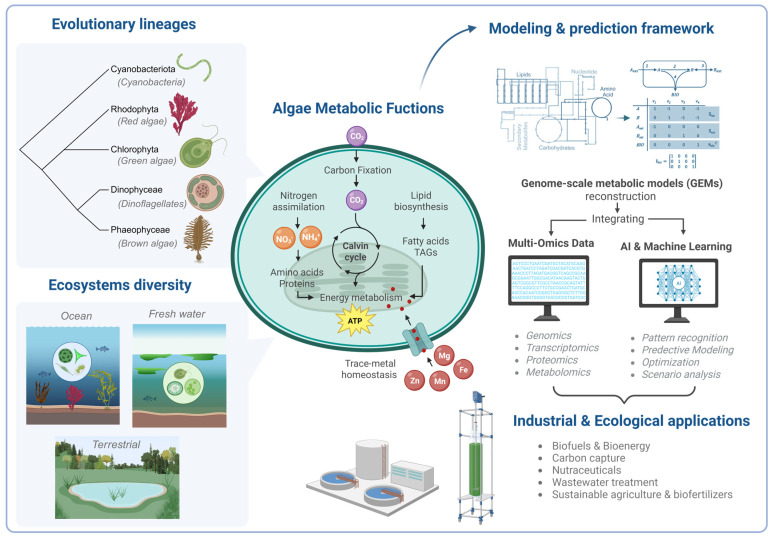
Integrative modeling of algal and cyanobacterial metabolism across diverse environments for industrial biotechnology.

Beyond their dominance in aquatic ecosystems, many algal lineages have successfully colonized terrestrial environments, forming essential components of soils, rocks, and even plant-associated microbiomes [6]. Algae from the phyla Chlorophyta, Streptophyta, Bacillariophyta, and Cyanobacteria serve as critical biological engineers of terrestrial ecosystems (Figure 1). These terrestrial algae initiate primary succession on barren substrates, secrete mucilage and exopolysaccharides that stabilize soil particles, and foster the establishment of microbial and plant communities [7]. Within biological soil crusts, they contribute to carbon fixation, nitrogen input, and mineral solubilization, enhancing soil fertility, aggregation, and resilience in arid and agricultural landscapes. Many terrestrial and epiphytic algae also engage in symbiotic associations with plants, colonizing rhizospheres, leaf surfaces, and bark. By releasing phytohormones, siderophores, and bioavailable micronutrients, these algae promote root growth, nutrient uptake, and the recycling of essential elements such as iron, zinc, and phosphorus [8]. Moreover, terrestrial algae synthesize unique stress-protective metabolites, including mycosporine-like amino acids, extracellular polysaccharides, and antioxidant pigments, that enable survival under desiccation, UV radiation, and extreme temperature fluctuations [9]. The total diversity of algae (i.e., marine, freshwater, and terrestrial) constitutes a metabolic scaffold that sustains ecosystem productivity, nutrient redistribution, and global biogeochemical stability [6].

Evolution of algae has refined sophisticated photosynthetic machinery, developing pigment composition and light-harvesting complexes to capture photons across diverse spectral and spatial gradients [10]. Chlorophylls, carotenoids, and phycobiliproteins act as complementary antenna pigments, enabling efficient photon capture from the intense blue wavelengths of the upper ocean to the far-red light of benthic environments [4]. This photoadaptive flexibility is paired with remarkable metabolic and physiological resilience. Algae have evolved to thrive under physicochemical extremes such as high salinity, fluctuating temperature, intense irradiation, and nutrient limitation employing specialized mechanisms (e.g., osmolyte synthesis, ionic regulation, and membrane remodeling) [11,12]. Equally remarkable is their ability to recycle and conserve essential nutrients, allowing them to persist in oligotrophic waters where other organisms fail. Algae developed high-affinity uptake systems for nitrogen, phosphorus, and sulfur, along with “luxury storage” mechanisms that accumulate nutrients during abundance and remobilize them during scarcity. Many algal lineages maintain close mutualistic relationships with bacteria, exchanging dissolved organic carbon, vitamins, and trace metals to enhance nutrient assimilation, detoxification, and metabolic efficiency. This dependence extends to the cycling and use of trace elements, which serve as indispensable cofactors in photosynthesis, respiration, nitrogen metabolism, antioxidant defense, and carbon fixation. Iron (Fe) drives electron transfer in photosystems I and II, cytochromes, and ferredoxins, directly linking metal homeostasis to photosynthetic efficiency and nitrogen assimilation. Manganese (Mn) constitutes the catalytic core of the oxygen-evolving complex of photosystem II, while magnesium (Mg) stabilizes chlorophyll molecules and facilitates photon absorption [13]. Copper (Cu) participates in photosynthetic and respiratory electron transport through plastocyanin and cytochrome *c* oxidase and also contributes to antioxidant protection through Cu/Zn-superoxide dismutases. Zinc (Zn) functions in carbonic anhydrases and other metalloenzymes, supporting CO_2_ hydration and carbon-concentrating mechanisms. Nickel (Ni) activates urease, permitting the utilization of urea as a nitrogen source. Cobalt (Co) is required for the synthesis of cobalamin (vitamin B_12_), a key cofactor for methyl-transfer and rearrangement reactions, and many algal species depend on bacterially supplied vitamin B_12_ for normal growth. Molybdenum (Mo) is required by molybdoenzymes such as nitrate reductase, supporting nitrate assimilation, and is also a component of nitrogenase in diazotrophic cyanobacteria, linking Mo availability to nitrogen acquisition and biological nitrogen fixation. Selenium, commonly available as selenite, contributes to redox homeostasis through selenoproteins such as glutathione peroxidases (Figure 1) [14]. These trace elements sustain the enzymatic framework underlying the Calvin–Benson cycle, carbon fixation, redox balance, and energy conversion. Through tight metabolic regulation and intracellular recycling of these elements, algae maintain high productivity and metabolic flexibility even under severe nutrient limitation [15].

The ability to redirect carbon toward energy-dense storage compounds is one of the most defining metabolic hallmarks of algae. Across evolutionary lineages, algae have diversified their lipid metabolism into a wide spectrum of structural and storage molecules, reflecting both their phylogenetic history and environmental adaptation [16]. The primary categories include neutral lipids, such as triacylglycerols (TAGs) and wax esters that serve as high-energy carbon reserves; polar or membrane lipids, including glycolipids and phospholipids that sustain photosynthetic and cellular membranes; and specialized secondary lipids, such as long-chain hydrocarbons, alkenones, sterols, and polyunsaturated fatty acids (PUFAs) with signaling, protective, and ecological roles. Unlike higher plants or most bacteria, many algal species are capable of producing unusually high proportions of long-chain and highly unsaturated fatty acids, such as eicosapentaenoic acid, docosahexaenoic acid, and arachidonic acid, which are essential to aquatic food webs and to human nutrition [17]. Diatoms, for example, channel carbon through desaturase–elongase pathways that efficiently generate C20–C22 PUFAs, whereas green algae predominantly synthesize C16 and C18 fatty acids for membrane homeostasis and energy storage. In contrast, red algae accumulate galactolipids rich in 20:4 and 20:5 fatty acids that maintain thylakoid fluidity under high salinity, and certain eustigmatophytes (e.g., *Nannochloropsis*) produce extremely high TAG yields exceeding 60% of their dry weight under nitrogen starvation levels, rarely matched by terrestrial plants or fungi [18]. This metabolic efficiency is sustained by specialized enzymatic machinery encoded in algal genomes, often expanded or reorganized through lineage-specific gene duplication and horizontal gene transfer. Core enzymes such as acetyl-CoA carboxylase, fatty acid synthase, and diacylglycerol acyltransferases form the backbone of de novo fatty acid and TAG biosynthesis. Additional algae-specific innovations include chloroplast-localized glycerol-3-phosphate acyltransferases and monoacylglycerol acyltransferases that couple photosynthetically derived glycerol with acyl chains, and lipid droplet-associated proteins that stabilize the resulting oil bodies. In thraustochytrids and certain diatoms, polyketide synthase-like complexes catalyze the stepwise formation of long-chain PUFAs through an oxygen-independent mechanism, representing one of the most efficient lipid-synthesizing systems known in nature. Many microalgae also encode lipid remodeling enzymes that respond dynamically to stress, reallocating fatty acids from membrane lipids to neutral storage forms when growth is limited by nitrogen, phosphorus, or light [19]. The roles of lipids in algae extend far beyond energy storage. Structural lipids such as mono- and digalactosyldiacylglycerol, as well as sulfoquinovosyldiacylglycerol are integral to thylakoid membranes, optimizing photosystem organization and electron transport under variable light intensities. Certain nonpolar lipids form hydrophobic surface layers that shield cells from desiccation, ultraviolet radiation, or metal toxicity, while carotenoid-containing lipid droplets contribute to antioxidant defense and photoprotection. PUFAs and oxylipins function as signaling molecules that mediate responses to stress and intercellular communication. They can act as chemical cues in predator–prey or symbiotic interactions within the planktonic community. In some bloom-forming diatoms, lipid-derived secondary metabolites modulate microbial competition or deter grazing, illustrating how lipid metabolism integrates into ecological fitness.

This evolutionary and ecological legacy has revealed algae as ecological keystone species and, from a human perspective, has established many algae as biotechnological platform organisms. The same metabolic versatility that enables survival in extreme and resource-limited environments also makes algae an exceptional living factory, capable of converting light energy, carbon dioxide, and wastewater nutrients into valuable bioproducts. Their rapid growth, photosynthetic efficiency, and diverse biochemical repertoire make them a sustainable chassis for industries focused on biofuels, pigments, nutraceuticals, and environmental remediation [20]. Understanding how evolution sculpted algal metabolism thus provides not only a window into the dynamics of aquatic ecosystems but also a blueprint for harnessing and optimizing their potential at an industrial scale.

The extraordinary biochemical and ecological diversity of algae presents both an opportunity and a challenge. While it underpins their global success, it also makes their metabolism difficult to predict and manipulate. To disentangle this complexity, modern research has turned toward systems biology frameworks that merge molecular data, network reconstruction, and computational prediction. Genome-scale metabolic models (GEMs) have become central to this effort, providing a quantitative scaffold that connects algal genomes to their physiological and ecological functions [21]. By integrating stoichiometric information, enzyme–gene associations, and compartmentalized reactions, GEMs enable the simulation of intracellular fluxes under different light regimes, nutrient availability, and environmental stresses. GEMs’ predictive capacity and robustness are greatly enhanced when coupled with multi-omics datasets, including transcriptomics, proteomics, metabolomics, and fluxomics [22]. Omics integration enables contextualization of the model and thereby reveals condition-specific metabolic states that cannot be inferred from genome annotation alone. With these systems biology approaches, researchers can now reconstruct environmentally responsive metabolic networks, capturing diel cycles, nutrient competition, and adaptive stress responses. Context-specific models also provide a quantitative bridge between cellular and ecological scales, allowing the simulation of algal interactions with bacteria and other planktonic organisms, and helping to infer community-level nutrient exchanges and competition for trace elements.

Simultaneously, the rapid expansion of algal genomic and environmental datasets has opened the door for Machine Learning (ML) and Artificial Intelligence (AI) to complement traditional constraint-based modeling. ML algorithms can uncover hidden patterns in high-dimensional omics and environmental data, identify regulatory modules controlling lipid accumulation or nutrient uptake, and predict growth phenotypes across light, temperature, and salinity gradients [23]. Recent advances in hybrid modeling frameworks, in which neural networks or evolutionary algorithms are embedded within stoichiometric models, now enable dynamic predictions of flux distributions and adaptive metabolic states with minimal manual curation [24].

In this review, we highlight how omics tools, GEMs, multi-omics integration, and AI-driven prediction have independently advanced our understanding of algal systems. We summarize how GEMs have been successfully employed to predict and unravel algal adaptation, and optimize targeted metabolites with industrial value, how omics studies have revealed molecular responses to environmental variability, and how ML and AI tools have optimized cultivation and trait prediction at an industrial scale. Yet, despite their individual successes, these disciplines have largely evolved in parallel, addressing either ecological or biotechnological questions in isolation [22]. We propose that the next major step in algal systems biology is the integration of these complementary frameworks into a cohesive paradigm that unites mechanistic modeling, data-driven inference, and environmental context. By combining the mechanistic rigor of GEMs, the contextual depth of omics, and the predictive capacity of AI, researchers can achieve a new level of understanding capable of revealing the mechanistic, dynamic, and predictive principles that govern algal metabolism, ecological roles, and industrial performance [21,25].

Representative habitats, including lake, soil, ocean, and tundra, are shown as ecological sources of diverse photosynthetic organisms, including green algae, red algae, brown algae, dinoflagellates, and cyanobacteria. GEMs, multi-omics data, and artificial intelligence can be integrated to predict algal metabolic capabilities and guide biotechnology applications. These approaches can support optimization of carbon fixation, nitrogen assimilation, lipid metabolism, and production of triacylglycerols (TAGs) and polyunsaturated fatty acids (PUFAs) under defined nutrient and cultivation conditions, including nitrogen, CO_2_, and trace metals such as Fe, Mn, Zn, and Mg.

## 2. From Genes to Function: Multi-Omics Insights into Algal Adaptation and Metabolic Regulation

**Figure 2:**
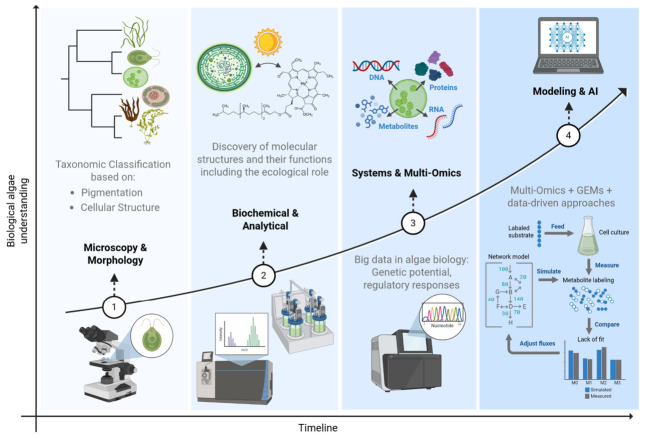
Historical progression of algal research from observational biology to predictive systems frameworks.

Early on, scientists used light microscopy to describe cell morphology, pigmentation, reproduction, and colony organization, gradually realizing that “algae” represented a polyphyletic assemblage of photosynthetic organisms rather than a single taxonomic group. This time period (the mid-1600s to the early 1900s) was known as the observation era (Figure 2). As analytical chemistry developed, pigment spectroscopy and thin-layer chromatography revealed the distinctive optical signatures of chlorophylls, carotenoids, and phycobiliproteins, connecting pigment composition to light-harvesting strategies and ecological niches [26]. In parallel, culture-based approaches revolutionized algal research: isolation in defined media, photobioreactor cultivation, and physiological assays allowed researchers to quantify photosynthetic rates, nutrient uptake, and growth kinetics under controlled conditions [27]. Before the rise of molecular biology, taxonomic and ecological differentiation relied on biochemical fingerprinting methods such as protein and isoenzyme electrophoresis, lipid and fatty-acid methyl ester (FAME) profiling, and polysaccharide composition analyses, which served as early molecular markers of strain and species identity [28]. Complementary experimental techniques, such as pigment ratios, nutrient utilization profiles, and light–dark oxygen-evolution measurements, provided the first multidimensional studies of algal physiology and diversity across environments. Remote sensing and pigment-based ocean-color mapping later expanded this view from laboratory cultures to complete ecosystems, establishing algae as major contributors in the biogeochemical cycles across both aquatic and terrestrial environments [29].

The emergence of molecular biology and genomics marked a turning point in our ability to unravel algal diversity and evolution. The introduction of DNA-based methods, including restriction-fragment length polymorphism (RFLP), random amplified polymorphic DNA (RAPD), amplified ribosomal DNA restriction analysis (ARDRA), and later 16S/18S rRNA sequencing and molecular phylogenetics during the late 1980s and 1990s, provided the first objective frameworks to identify algal taxa and understand their evolutionary relationships (Figure 2). During the 1990s and early 2000s, these molecular approaches revealed that many morphologically similar algae actually represented genetically distinct lineages, exposing a hidden layer of biodiversity that had remained undetected by previous scientific methods. At the same time, molecular evidence also uncovered evolutionary connections between eukaryotic algae and cyanobacteria, providing strong support for ancient endosymbiotic events that led to the origin of modern chloroplasts and the diversification of photosynthetic mechanisms. By the early 2000s, as sequencing technologies advanced, whole-genome sequencing began to transform algal biology from a primarily descriptive discipline into a mechanistic science.

Algal research has evolved through four major conceptual and technological eras that progressively increased biological resolution and mechanistic understanding. (1) The Microscopy and Morphology Era established early taxonomic classification based on pigmentation, cellular architecture, and colony organization, revealing the structural diversity of algal lineages. (2) The Biochemical and Analytical Era linked molecular composition to ecological function through pigment spectroscopy, chromatography, lipid profiling, and nutrient utilization assays, connecting metabolites and biomolecules to physiological strategies. (3) The Systems and Multi-Omics Era introduced genome sequencing, transcriptomics, proteomics, and metabolomics, enabling high-throughput characterization of genetic potential, regulatory responses, and metabolic states across environmental conditions. This transition marked the emergence of “big data” biology in algae. (4) The Modeling and Artificial Intelligence Era integrates multi-omics datasets with genome-scale metabolic models (GEMs) and data-driven approaches, enabling predictive simulations of metabolic fluxes, stress responses, and ecological interactions.

As sequencing technologies matured, whole-genome sequencing transformed algal biology from descriptive taxonomy into a mechanistic science. Genomic analyses of model species such as *Chlamydomonas reinhardtii* [30], *Phaeodactylum tricornutum* [31], and *Nannochloropsis gaditana* [32] unraveled the gene families and enzymatic functions that enabled adaptation to diverse habitats: carbon-concentrating mechanisms, metal-binding proteins for trace-element acquisition, desaturases and polyketide synthases for lipid diversification, and stress-responsive regulators under different biotic and abiotic stressors. Environmental genomics and barcoding expanded this framework to natural communities, revealing that algal diversity extends from deep oceans and glacial ice to soils, rocks, and plant-associated microhabitats [33]. These initial genomic efforts not only cataloged the taxonomic and genetic breadth of algae but also laid the foundation for a new phase of research focused on connecting genomic information to functional, ecological, and evolutionary processes [34].

The appearance of functional, comparative, and environmental genomics transformed algal research from a cataloging discipline into a predictive molecular science, as new genomes are characterized (Figure 2) [35]. Functional genomics linked genome content to physiological traits through mutagenesis, reverse genetics, and expression profiling, revealing the genetic basis of photosynthetic efficiency, nutrient assimilation, and lipid metabolism in key species (*C. reinhardtii*, *P. tricornutum*, *C.* and *N. gaditana*). Comparative genomics revealed that horizontal gene transfer, gene duplication, and neofunctionalization have shaped algal evolution. The sequencing of streptophyte algae such as *Klebsormidium nitens*, *Zygnema circumcarinatum*, and *Penium margaritaceum* revealed that many genes once considered exclusive to land plants (regulators of phytohormone signaling, desiccation tolerance, and morphogenesis, etc.) have deep algal ancestry, redefining the molecular origins of terrestrial adaptation [36]. Similarly, the chimeric genomes of diatoms and brown algae indicate that extensive acquisition of bacterial and archaeal genes underlies their ecological success in dynamic marine environments [37]. Metagenomic sequencing extended this genomic exploration to entire ecosystems, elucidating the algal diversity in oceans, freshwater bodies, and soils [38]. For instance, environmental assemblies of glacier algae (*Ancylonema*), coral symbionts (*Symbiodiniaceae*) revealed lineage-specific strategies for micronutrient recycling, nitrogen fixation, and photoprotection under stress [39]. The growing accessibility of high-quality genome assemblies through initiatives such as the DOE JGI’s PhycoCosm [40], which integrates more than 250 algal genomes, and global sequencing efforts like 10KP [41] has established a comprehensive genomic infrastructure for cross-lineage comparisons and ecological modeling. The development of algal-focused bioinformatic resources has become essential for organizing and interpreting the expanding information about new genomes and metagenome collections [42]. Beyond extending our understanding of algal evolution and ecological function, genomics tools have also elucidated the vast biotechnological potential encoded within algal genomes [26]. Genome annotations have revealed key enzymatic pathways involved in lipid biosynthesis, pigment production, vitamin metabolism, and carbon fixation, enabling the identification of promising species and strains for biofuel generation, carbon capturing, and sustainable bioproduct synthesis. Consequently, genomics has expanded the role of algal research from evolutionary and ecological inquiry to a foundation for bioprospecting and metabolic engineering aimed at industrial and environmental innovation [43].

While genomics and metagenomics have revolutionized our ability to catalog algal diversity and predict metabolic potential, they inherently provide only a static view of the biological landscape. Genome sequences provide insights into an organism’s potential, but not necessarily what function it carries out under specific environmental conditions. Genomic information alone cannot explain how algae dynamically regulate their physiology to survive and adapt to changing habitats, nor can it resolve how nutrients are assimilated, transformed, and recycled within their ecosystems [44]. The genetic code outlines potential biochemical capacities but offers limited insight into gene expression patterns, protein abundance, metabolite production, or molecular interactions that define an organism’s real-time functional state. Since algal organisms are constantly exposed to fluctuations of multiple biotic and abiotic factors such as light intensity, water abundance, presence of other microorganisms, salinity, temperature, and nutrient availability, it is essential to understand how metabolic networks respond to environmental stressors, modulate energy allocation, and sustain ecological roles. To move from metabolic potential to function, the field turned to the next generation of omics tools, generating a multidimensional framework capable of capturing the mechanistic basis of algal adaptation and metabolic regulation across environmental contexts [45].

Transcriptomics have provided a dynamic view of algal function, revealing how gene expression patterns shift in response to changing environments and developmental cues [46]. Across marine, freshwater, and soil algae, RNA-seq analyses have uncovered the molecular plasticity that underpins resilience to environmental stress [47,48]. In diatoms such as *P. tricornutum*, nutrient deprivation and high salinity induce coordinated upregulation of lipid biosynthetic genes (DGATs, PDATs) and downregulation of nitrogen assimilation and photosynthetic transcripts, reallocating carbon toward triacylglycerol accumulation without severely compromising growth [49]. Similarly, *C. reinhardtii* exhibits transcriptional reprogramming of carbon-concentrating mechanisms and antioxidant enzymes under high CO_2_ or light stress, maintaining redox balance and photoprotection (Table 1) [50]. In the brown alga *Ectocarpus siliculosus*, transcriptomics revealed that osmotic and oxidative stresses activate a shared core of stress-responsive genes encoding heat-shock proteins, transporters, and ROS-scavenging enzymes, supporting rapid acclimation to intertidal fluctuations [47]. Comparative studies in the red alga *Porphyra umbilicalis* and the streptophyte *Klebsormidium nitens* identified convergent transcriptional strategies for desiccation tolerance, including induction of LEA proteins, late-embryogenesis protectants, and trehalose biosynthetic genes. Transcriptome profiling has also clarified algal ecological interactions: in coral–algal symbioses, expression of coral immune genes is downregulated while endocytosis and carbon metabolism genes are upregulated during the establishment of *Symbiodinium* symbionts, highlighting molecular cross-talk between host and alga [51]. More recently, single-cell RNA sequencing of brown algae such as *Fucus serratus* revealed a “transcriptomic hourglass,” in which evolutionarily conserved gene networks dominate mid-developmental stages, paralleling patterns in animals and plants [52]. Furthermore, transcriptomic analyses have also driven advances in algal biotechnology. Expression profiling under different cultivation regimes has guided the optimization of light, temperature, and nutrient inputs to enhance lipid, carotenoid, and pigment accumulation in industrially relevant species such as *Nannochloropsis*, *Dunaliella*, and *Haematococcus* [53]. Differential expression analyses have identified transcription factors and enzymes regulating astaxanthin, β-carotene, and fatty acid biosynthesis, enabling targeted metabolic engineering to increase yields [53]. Transcriptome-guided discovery of enzymes with enhanced thermostability, halotolerance, or substrate specificity has accelerated the development of algal biocatalysts for biofuel, pharmaceutical, and food industries [47].

Proteomics provides a crucial bridge between genomic potential and physiological reality by directly quantifying the protein complement of a cell, thereby revealing the molecular machinery actively executing metabolic functions [54]. Unlike genomics and transcriptomics, which describe gene content and expression potential, proteomics captures the actual protein abundance, turnover, subcellular localization, and post-translational modifications that determine enzyme activity and metabolic flux [27,55]. However, proteomes are vastly more complex than genomes due to the coexistence of thousands of proteins spanning several orders of magnitude in abundance and numerous proteoforms generated by post-translational modifications such as phosphorylation, acetylation, glycosylation, and ubiquitination [54]. Modern algal proteomics spans expression, functional, and structural branches: expression proteomics tracks abundance changes across environmental conditions; functional proteomics identifies enzyme activities and interaction partners; and structural proteomics characterizes complex assemblies such as photosystems and carbon-concentrating mechanisms [27,55]. In *P. tricornutum*, quantitative proteomics under different CO_2_ levels revealed metabolic reorganization between C_3_ and C_4_-like pathways, demonstrating flexible carbon fixation strategies that balance energy supply and nitrogen assimilation under variable inorganic carbon availability [56]. In *Lobomonas rostrata*, isobaric-tag proteomics uncovered the metabolic trade-offs required to sustain a mutualistic association with *Mesorhizobium loti*, including upregulation of amino acid biosynthesis and stress-response proteins coupled with downregulation of photosynthetic complexes, which is a direct evidence of protein-level adaptation to symbiosis [57]. Proteomic analyses have also elucidated nutrient-dependent regulation in *Chlorella vulgaris* and *Scenedesmus obliquus*, where nitrogen limitation triggered induction of lipid-assembly enzymes and suppression of photosynthetic proteins, explaining carbon redirection toward triacylglycerol synthesis [58]. Furthermore, proteomics now reinforce industrial optimization of algal strains: large-scale quantitative studies identify rate-limiting enzymes for lipid, carotenoid, and protein production; phosphoproteomics reveals regulatory nodes for enhancing stress tolerance and biomass yields; and food-oriented proteomic surveys guide the selection of species with high digestibility and balanced amino-acid profiles for human nutrition [59].

While proteomics connects gene expression to protein abundance and activity, metabolomics provides the final functional readout of cellular physiology, revealing the small molecules that reflect the real-time metabolic state of algal organisms [60,61]. Unlike genomics, which describes potential, or transcriptomics and proteomics, which reveal molecular regulation and protein dynamics, metabolomics captures the downstream outcome of all cellular processes, offering a biochemical fingerprint of how algae interact with and respond to their environment [60]. The identification of intracellular and extracellular metabolites across time and conditions enables the identification of active metabolic pathways and the quantification of flux changes that occur in response to environmental fluctuations. Both targeted and untargeted metabolomics approaches have been instrumental in algae to reveal how light intensity, nutrient limitation, salinity, and temperature reshape the metabolome without necessarily reducing growth performance [62,63]. Untargeted metabolomics in marine and freshwater algae has shown the release of signaling molecules, amino acids, peptides, and lipophilic compounds that mediate competition, nutrient exchange, and microbial interactions, revealing the chemical dimension of algal ecology [63,64]. In aquatic ecosystems, metabolite profiling has also been applied to monitor eutrophication, nutrient depletion, and the production of allelopathic or toxic compounds that modulate microbial community structure [64]. In the industrial context, metabolomics has become essential for identifying stress-induced metabolic switches that trigger the accumulation of bioactive molecules, pigments, and storage lipids [65,66]. Targeted and untargeted lipidomics have mapped how algae remodel their membrane composition and carbon allocation under nutrient deprivation, enabling the optimization of cultivation parameters for large-scale production of high-value fatty acids, carotenoids, and antioxidants [66,67].

Emergent omics approaches are now expanding the frontier of our mechanistic understanding. One of the most powerful among them is ribosome profiling (Ribo-seq), which captures ribosome-protected mRNA fragments to provide a high-resolution view of translation in action [68]. Unlike transcriptomics, which measures transcript abundance, ribosome profiling reveals which transcripts are actively translated and at what efficiency, uncovering layers of post-transcriptional regulation that define the proteome [69]. Recent studies in *C. reinhardtii* employing optimized Ribo-seq protocols have resolved translational dynamics across the nuclear, chloroplast, and mitochondrial genomes, revealing distinct elongation rates and regulatory pauses associated with photosynthetic complexes [69]. These analyses have also identified upstream open reading frames (uORFs) and alternative initiation sites that modulate gene expression during diurnal cycles and stress responses, providing unprecedented insight into how translation contributes to circadian and environmental adaptation [69,70]. Beyond ribosome profiling, other emerging omics such as interactomics and fluxomics are beginning to bridge remaining gaps between molecular data and functional systems. Interactomics maps the physical and regulatory connections among proteins, metabolites, and nucleic acids that coordinate photosynthetic efficiency and stress tolerance, while fluxomics quantifies intracellular carbon, nitrogen, and energy flow to reveal how these interactions shape metabolic performance [71]. Despite these advances, studies applying novel omics approaches to algae remain limited compared to those in plants or bacteria. Expanding their use will be essential to capture the full regulatory complexity of algal cells, uncover novel mechanisms that sustain their environmental adaptability, and build comprehensive datasets that integrate translational, interaction, and flux-level information across diverse taxa. Increasing efforts in this direction will not only refine our understanding of algal systems biology but also unlock new opportunities for ecological prediction and industrial innovation.

When individual omics approaches are applied in isolation, they provide snapshots of specific molecular layers (e.g., transcriptional reprogramming or metabolite accumulation) that can be correlated with physiological changes [71]. However, these correlations often fall short of explaining the underlying mechanisms linking genotype to phenotype [66]. Integrating multiple omics technologies overcomes these limitations by enabling multilayer analyses that capture concurrent changes across transcripts, proteins, and metabolites under defined environmental or developmental conditions [72,73]. Multi-omics frameworks allow the reconstruction of causal relationships among molecular components and provide temporal and spatial resolution to complex regulatory events [72,74]. For instance, combined transcriptomic, phosphoproteomic, and functional genomics profiling of *C. reinhardtii* under osmotic stress revealed how cytoskeletal organization, ion transport, and MAP kinase signaling jointly sustain water homeostasis across cellular compartments [73]. Similarly, integrated genome, transcriptome, and metabolome analyses of the halotolerant *Scenedesmus* sp. NREL 46B-D3 uncovered coordinated regulation of fatty acid biosynthesis and membrane lipid remodeling under temperature stress, identifying transcription factors and metabolic pathways that stabilize growth and lipid accumulation in suboptimal conditions [74]. These and other studies demonstrate that multi-omics integration moves algal research beyond descriptive correlations, allowing mechanistic interpretation of how molecular networks reorganize to maintain homeostasis and productivity [72,75]. As more datasets become available, multilayer omics will be pivotal to building predictive models of algal metabolism and to guiding rational engineering for ecological resilience and industrial optimization (Table 1).

Omics technologies have been particularly valuable for resolving the regulation of high-value metabolic pathways in algae, including carotenoid, xanthophyll, ketocarotenoid, and lipid biosynthesis. These pathways are strongly influenced by cultivation and environmental parameters such as nitrogen availability, light intensity, salinity, temperature, pH, CO_2_ supply, and trophic regime. Rather than acting as isolated product-forming routes, they function as environmentally responsive carbon-allocation modules that balance growth, photoprotection, redox homeostasis, membrane remodeling, and storage metabolism. For example, nitrogen limitation frequently reduces protein synthesis, chlorophyll biosynthesis, and photosynthetic membrane expansion, thereby redirecting carbon skeletons and reducing power toward TAGs or secondary pigments. High light and salinity impose additional photooxidative and osmotic stress, activating carotenoid-based protective responses and altering the partitioning of carbon between biomass formation, storage lipids, and antioxidant compounds. Thus, omics-based studies have provided a mechanistic framework to understand how algal cells convert environmental stress into pathway-specific metabolic outputs.

Ketocarotenoid biosynthesis has been studied most extensively in *H. pluvialis* and *Chromochloris zofingiensis*, two green algae capable of accumulating astaxanthin under stress. In *H. pluvialis*, genome and transcriptome sequencing identified genomic resources and candidate genes involved in astaxanthin synthesis, accumulation, and regulation, including the conversion of β-carotene toward astaxanthin through β-carotene ketolase and β-carotene hydroxylase activities. The transition from motile green cells to red immobile cysts is associated with stress conditions such as high irradiation, salinity, and nutrient deprivation, which induce cell enlargement, loss of flagella, thickened cell walls, and astaxanthin accumulation [76]. Metabolomics and network analyses further showed that combinations of acetate, Fe^2+^, and high light reshape central metabolism during astaxanthin accumulation. GC–MS and LC–MS profiling identified 93 stable and 24 unstable intracellular metabolites, while WGCNA resolved stress-associated metabolic modules linked to high light, Fe^2+^ plus high light, and acetate plus Fe^2+^ plus high light conditions. The same study identified α-ketoglutarate, glutamate, and ribose-5-phosphate as metabolites associated with Fe^2+^ plus high light, suggesting that astaxanthin induction is coupled to nitrogen–carbon metabolism, amino acid metabolism, and pentose phosphate pathway activity [77]. Transcriptomics analysis under high light–sodium acetate stress added a regulatory layer by identifying 4367 differentially expressed genes and 69 differentially expressed transcription factors; six TFs from MYB, MYB_related, NF-YC, Nin-like, and C3H families were predicted to regulate 27 astaxanthin-related genes, with CrtO emerging as a central regulatory hub [78].

β-carotene biosynthesis in *Dunaliella salina* provides a complementary example in which carotenoid accumulation is tightly connected to salinity tolerance, high-light acclimation, and osmotic regulation [79]. Genomic and transcriptomic analyses of *D. salina* FACHB435 generated a 472 Mb genome with 30,752 predicted protein-coding genes and showed that high light promotes β-carotene accumulation during culture. Differential expression analysis identified upregulation of carotenoid pathway genes including DsCrtB, DsPDS, DsZ-ISO, DsZDS, DsCRTISO, DsLUT5, DsCrtL-B, and DsCCD8, whereas DsCrtF and DsLUT1 were downregulated, highlighting coordinated transcriptional remodeling of the carotenoid pathway under light stress [53]. Functional characterization of β-carotene biosynthetic genes in *Dunaliella* further clarified the enzymatic logic of the pathway by confirming the roles of GGPS, PSY, PDS, ZISO, ZDS, CRTISO, and LYCB in the conversion from geranylgeranyl pyrophosphate to β-carotene. This work also showed that the algal route from phytoene to lycopene resembles the plant-type pathway, requiring PDS, ZISO, ZDS, and CRTISO, rather than relying on a bacterial-type CrtI-like single-enzyme desaturation system [80]. Importantly, transcriptional activation does not always translate linearly into β-carotene accumulation. In *D. salina* GY-H13, β-carotene increased under nitrogen deficiency, Cd exposure, and high light, but decreased under high salinity; early stress responses activated different subsets of β-carotene genes, while later time points showed partial mismatches between transcript levels and pigment accumulation. This indicates that β-carotene production is regulated not only transcriptionally but also through timing, stress intensity, enzyme activity, plastid redox state, and possibly post-transcriptional or post-translational regulation.

Xanthophyll and lipid pathways illustrate how algae remodel photosynthesis and storage metabolism in response to light and nutrient availability. In *P. tricornutum*, fucoxanthin biosynthesis is closely linked to fucoxanthin–chlorophyll a/c binding protein (FCP) complexes in the thylakoid membrane. Under low light, fucoxanthin content and productivity reached 1.7 mg g^−1^ and 2.12 mg L^−1^ day^−1^, respectively, whereas high light reduced these values to 0.54 mg g^−1^ and 0.79 mg L^−1^ day^−1^. Proteomic analysis of thylakoid membrane fractions and transcriptomic analysis showed enrichment of FCP antennae under low light and identified PtLhcf5 and PtLhcf8 as important components of FCP biosynthesis, while high light promoted FCP degradation. Other light-acclimation studies in *P. tricornutum* showed that high light induces rapid regulation of photosynthesis, pigment metabolism, ROS-scavenging systems, and photoprotective metabolites within the first 0.5 h, followed by later remodeling of the photosynthetic apparatus [81]. The diatom xanthophyll cycle is also regulated by the redox state of the plastoquinone pool, which controls LHCX expression, diadinoxanthin/diatoxanthin dynamics, and energy-dependent quenching, supporting plastid-to-nucleus retrograde signaling during high-light acclimation. Lipid biosynthesis follows a related stress-responsive logic [82]. In *N. gaditana*, nitrogen starvation increased TAG accumulation to 38% dry weight, mainly through de novo fatty acid synthesis, while chloroplast galactolipids decreased and the photosynthetic apparatus was reorganized through reductions in PSII, PSI, and cytochrome b_6_f, compensated partly by cyclic electron flow [83]. In *P. tricornutum*, integrated transcriptome, proteome, and metabolome profiling under nitrogen limitation revealed that 22% of transcripts, 17% of proteins, and 44% of metabolites were differentially regulated; nitrogen limitation upregulated central carbon metabolism and TCA-cycle-associated processes while downregulating photosynthetic and ribosomal protein synthesis, suggesting that TAG accumulation depends on both lipid remodeling and the availability of carbon flux through central metabolism [84]. Omics findings have established that fatty acid and TAG production are controlled by nutrient-dependent carbon overflow, photosynthetic reorganization, lipid remodeling, DGAT/lipase activity, and lineage-specific partitioning between TAG, membrane lipids, EPA-rich lipids, and storage carbohydrates.

The transition from data generation to predictive understanding represents the next frontier in algal research. The accumulation of multi-omics datasets enables the development of systems biology frameworks that integrate molecular, physiological, and ecological information into coherent models of algal function [85]. Connecting experimental evidence with computational modeling allows the simulation and prediction of how environmental or genetic perturbations propagate through cellular networks to influence growth, metabolism, and community interactions. GEMs are at the core of this effort, translating genomic and biochemical knowledge into quantitative representations of metabolic fluxes that can be validated and refined using transcriptomic, proteomic, and metabolomic data. Integrative modeling has begun to elucidate how algae balance resource allocation under stress, coordinate photosynthetic and respiratory metabolism across light–dark cycles, and optimize nutrient uptake and energy conversion in dynamic ecosystems [86]. Besides providing mechanistic insight, systems biology frameworks serve as predictive tools for metabolic engineering and cultivation optimization, enabling the design of strategies to enhance lipid yields, carbon fixation, or tolerance to salinity and nutrient limitation [87]. As the field advanced, combining experimental multi-omics with predictive modeling has been essential to bridge molecular detail with ecological and biotechnological applications (Figure 2).

## 3. Mechanistic Modeling in Algae: GEM Reconstruction, Curation, and Contextualization

Systems biology has transformed our capacity to study living organisms from a mechanistic and holistic perspective [88]. Rather than examining individual genes or reactions in isolation, systems biology integrates biochemical, genetic, and omics information into quantitative frameworks that describe how molecular networks shape physiology and adaptation [89]. At the center of this paradigm lies the development of GEMs which are comprehensive reconstructions that translate annotated genomes into stoichiometrically balanced networks of metabolic reactions [90]. These models unify information from multiple disciplines, combining biochemistry (reaction mechanisms and thermodynamics), genetics (gene–protein–reaction associations), omics sciences (transcriptomics, proteomics, metabolomics), and computational modeling to simulate how metabolism responds to environmental, genetic, or physiological perturbations [25]. The first GEMs were built for bacteria, such as *Haemophilus influenzae* [91], *Escherichia coli* [92], and *Bacillus subtilis* [93], which offered tractable systems due to their relatively small genomes, well-characterized metabolism, and abundant experimental data [94]. Early bacteria metabolic models validated the potential of constraint-based modeling to reproduce experimentally measured growth rates, gene essentiality, and substrate utilization patterns [89]. As sequencing technologies and annotation accuracy improved, the same principles were extended to eukaryotic systems [90]. Initial GEMs were built for unicellular fungi and yeasts, and later extended to more complex photosynthetic organisms such as algae and even plants [88]. Building GEMs for eukaryotes introduced new computational challenges, including the compartmentalization of reactions across organelles, the presence of isoenzymes and multi-subunit complexes, and even in some particular scenarios, the integration of regulatory and signaling layers [90]. Nonetheless, these advances set the basis for algal metabolic modeling, where multiple subcellular compartments (e.g., nucleus, Golgi apparatus, chloroplast, mitochondrion, peroxisome, cytosol, thylakoid lumen, etc.) interact through a fully connected metabolic network that balances energy production, redox homeostasis, and precursor biosynthesis to sustain growth [25].

**Figure 3:**
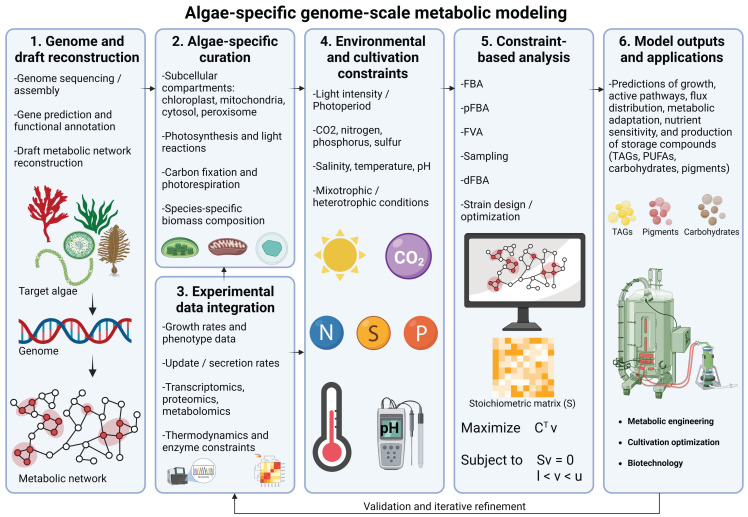
Algae-specific genome-scale metabolic modeling workflow.

Constraint-based metabolic modeling provides the theoretical foundation for simulating and analyzing these genome-scale networks. By defining a stoichiometric matrix that represents all known biochemical reactions in the organism, the system is solved under a pseudo–steady-state assumption, where intracellular metabolite concentrations remain constant over time [95]. Within this framework, optimization algorithms such as flux balance analysis (FBA) are employed to estimate feasible reaction flux distributions that maximize a biological objective function (BOF), typically the biomass production rate. Variants like parsimonious FBA (pFBA) minimize the total flux burden while preserving optimal growth, and flux variability analysis (FVA) identifies the permissible flux ranges consistent with the same objective, providing insight into metabolic redundancy and flexibility (Figure 3) [96]. Additionally, random sampling or Monte Carlo–based approaches explore the broader solution space, revealing alternative pathways and trade-offs under the imposed physicochemical constraints. Importantly, this solution space can be further refined by incorporating experimental or biological data, including thermodynamic bounds, metabolite uptake and secretion rates, enzyme abundances, and transcriptomic or proteomic expression profiles, to constrain reaction activity toward physiologically realistic conditions [97]. Integrating such data narrows the range of feasible metabolic states and allows the generation of context-specific models that more accurately reflect the dynamic regulation of metabolism under distinct environmental or experimental conditions [88,90]. The application of constraint-based modeling principles to photosynthetic organisms began with the reconstruction of *i*JN678, the first GEM of the cyanobacterium *Synechocystis* sp. PCC 6803 [98]. As a bacterial phototroph, *Synechocystis* represented an ideal starting point due to its relatively compact genome, well-characterized photosynthetic machinery, and central role as a model for oxygenic photosynthesis. The *i*JN678 model integrated 678 genes, 863 reactions, and 795 metabolites distributed across cytoplasmic, thylakoid, periplasmic, and extracellular compartments [99]. *i*JN678 successfully captured the coupling between light-driven electron transport, carbon fixation through the Calvin–Benson–Bassham cycle, and nitrogen assimilation, allowing quantitative prediction of growth under photoautotrophic, mixotrophic, and heterotrophic conditions. Beyond validating stoichiometric consistency, the model revealed metabolic trade-offs between photosynthetic ATP generation and respiratory flexibility, highlighting how cyanobacteria balance energy and redox demands across light and dark phases.

A wide ecosystem of computational resources now supports the reconstruction, curation, and analysis of algal genome-scale metabolic models. General-purpose modeling frameworks such as the COBRA Toolbox, COBRApy [100], RAVEN [101], merlin [102], AuReMe [103], ModelSEED [104], and KBase [105] provide automated and semi-automated pipelines for draft model construction, gap-filling, stoichiometric validation, and simulation, forming the backbone of most current algal reconstructions. Complementary biochemical and enzymatic databases, including KEGG [106], MetaNetX [107], Rhea [108], BRENDA [109], TransportDB [110], SwissLipids [111], and ChEBI [112], supply the reaction mechanisms, enzyme functions, transporter families, metabolite identifiers, and ontological mappings required to curate reaction directionality, compartment assignment, gene–reaction associations, and biomass compositions. Visualization environments such as Escher v 1.8.1 and Cytoscape v 3.10.4 facilitate pathway-level inspection and flux map generation, enabling researchers to identify inconsistencies, detect missing reactions, and propose new hypotheses about pathway organization [113]. Critically for algae, specialized resources such as PhycoCosm [40], PlantCyc [114], AlgaePath [115], MMETSP [116], and Ocean Gene [117] provide high-quality genomic, transcriptomic, and environmental datasets that support lineage-specific annotation and comparative metabolic analyses. These computational resources expand the modeling landscape, enabling workflows that integrate genomic annotation, biochemical evidence, multi-omics data, and environmental information into coherent mechanistic frameworks. As more algal genomes, transcriptomes, and metabolomes continue to accumulate, general and algal-specific bioinformatics databases will become central to standardizing model curation, enhancing annotation accuracy, and supporting cross-lineage comparisons that connect metabolic traits with ecological roles and biotechnological potential in algae [118].

The figure summarizes the major steps required to build, curate, constrain, analyze, and apply algae-specific GEMs. First, genome sequencing/genome assembly, gene prediction, and functional annotation are used to generate an initial draft metabolic network reconstruction for the target algae. Second, algae-specific curation incorporates key biological features such as subcellular compartmentalization, including chloroplast, mitochondria, cytosol, and peroxisome; photosynthesis and light reactions; carbon fixation and photorespiration; and species-specific biomass composition. Third, experimental datasets, including growth and phenotype measurements, nutrient uptake and secretion rates, transcriptomics, proteomics, metabolomics, thermodynamic information, and enzyme constraints, are integrated to refine the model. Fourth, environmental and cultivation constraints such as light intensity, photoperiod, CO_2_ availability, nitrogen, phosphorus, sulfur, salinity, temperature, pH, and trophic conditions are incorporated to simulate realistic growth scenarios. Fifth, constraint-based approaches, including FBA, pFBA, FVA, sampling, and dFBA are used to optimize biomass and predict flux distributions and active metabolic pathways. Finally, model outputs support prediction of growth, metabolic adaptation, nutrient sensitivity, and production of storage compounds such as TAGs, PUFAs, carbohydrates, and pigments, enabling applications in metabolic engineering, cultivation optimization, and algal biotechnology.

The development of iRC1080, the first comprehensive eukaryotic algal metabolic model for *C. reinhardtii*, represented a landmark in photosynthetic systems biology. Unlike cyanobacteria, *C. reinhardtii* possesses a complex intracellular architecture with distinct metabolic functions partitioned across the chloroplast, mitochondrion, peroxisome, cytosol, and thylakoid lumen [119]. The *i*RC1080 model encompassed 2190 reactions, 1080 genes, and 1068 unique metabolites distributed across ten compartments, and was the first to incorporate photon wavelength resolution into a constraint-based framework, updating the previous efforts of the AlgaGEM reconstruction [90]. The integration of prism reactions that translate light spectra into stoichiometric photon fluxes across effective wavelength ranges into iRC1080 enabled simulation of algal metabolism under diverse light sources, including solar, fluorescent, and LED illumination, capturing the quantitative relationship between spectral composition and growth efficiency.

Quantitative photon integration into modeling frameworks linked photon absorption to photosystem activity and pigment synthesis, providing a mechanistic description of how *C. reinhardtii* allocates absorbed light energy into biochemical processes. Besides accurately capturing *C. reinhardtii*’s photosynthetic mechanisms, iRC1080 expanded lipid metabolic pathways with unprecedented biochemical detail, enumerating stereochemical configurations, acyl-chain lengths, and double-bond positions of fatty acids and glycerolipids. This enabled reconstruction of latent and lost lipid pathways, offering evolutionary insights into the absence of very-long-chain and sphingolipid biosynthesis in *C. reinhardtii*. The model was validated both genetically and environmentally: more than 75% of the genes were experimentally confirmed by transcript verification, while growth simulations under photoautotrophic, mixotrophic, and heterotrophic conditions accurately reproduced measured phenotypes, including photosynthetic O_2_ evolution and energy conversion efficiency under different light regimes. Gene essentiality analyses identified metabolic subsystems critical under specific environmental conditions. For instance, *i*RC1080 captured photosynthesis and porphyrin metabolism under light, or glycolysis and starch degradation under dark conditions, demonstrating the model’s predictive capacity for genotype–phenotype relationships. Importantly, the light-source-specific validations revealed that model predictions closely matched experimental growth under red, white, and solar illumination, leading to a novel application: the in silico design of optimal LED spectra for maximal biomass yield and photon utilization efficiency. *i*RC1080 set the initial precedent of an algal metabolic model (excluding cyanobacteria) employed to both understand the metabolic mechanisms of *C. reinhardtii* under different environmental conditions and provide a computational tool to optimize the production of industrial high-value compounds (Table 2) [25].

Following the pioneering reconstruction of *C. reinhardtii*, algal metabolic modeling expanded to additional lineages with distinct evolutionary origins, physiology, and biotechnological potential [21,120]. *i*CZ843 for *C. vulgaris* and iLB1027_lipid for *P. tricornutum* elucidate how increasing biochemical complexity, expanded lipid catalogs, and richer experimental constraints shaped the next generation of algal GEMs [21,120]. Despite the substantial differences between green algae and diatoms, both models display a characteristic feature of eukaryotic phototrophs: a disproportionately large metabolic reaction space underpinned by a relatively small subset of metabolic genes, typically representing <15% of the total genome, yet supporting thousands of reactions distributed across chloroplasts, mitochondria, peroxisomes, the cytosol, specialized plastid subdomains, and other eukaryotic compartments. Both GEMs incorporated organism-specific BOFs with greater biochemical granularity than earlier reconstructions, including accurate distributions of proteins, carbohydrates, pigments, and lipid classes supported by FTIR spectroscopy, FAME profiling, and nutrient-limitation experiments. Detailed biomass compositions enabled simulations of dynamic phenotypes such as nitrogen starvation responses, lipid remodeling, and shifts in carbon allocation [121]. *i*CZ843 successfully reproduced experimental growth patterns under autotrophic, heterotrophic, and mixotrophic conditions and predicted metabolic rerouting associated with nitrogen limitation, carbon excess, and lipid accumulation [122]. Meanwhile, *i*LB1027_lipid underwent extensive validation against photoautotrophic phenotypes, diel metabolic patterns, nitrogen depletion experiments, and quantitative carbon partitioning measurements [123]. It captured hallmark diatom features, including the urea cycle, light-dependent carbon allocation, plastid–mitochondrial energetic coupling, and the emergence of a previously unrecognized glutamine–ornithine shuttle connecting redox and nitrogen metabolism. These reconstructions also introduced new applications in algal systems biology. *i*CZ843 was used to evaluate media design strategies, identify flux bottlenecks limiting lipid productivity, and guide metabolic engineering toward increased TAG yields [124]. *i*LB1027_lipid enabled in silico exploration of electron flow under variable light, prediction of neutral-lipid accumulation thresholds, and quantification of carbon partitioning between chrysolaminarin and triacylglycerols across irradiance levels [125]. Moreover, both models provided frameworks for metabolic engineering: *C. vulgaris* for sustainable biofuel feedstocks, and *P. tricornutum* for high-value lipids, omega-3 fatty acids, and bioproducts enabled by its unique evolutionary mosaic of bacterial, red algal, and secondary endosymbiotic genes (Table 2) [126].

Metabolic modeling efforts rapidly expanded across the major eukaryotic algal lineages, producing a diverse portfolio of reconstructions that capture lineage-specific biochemistry, photophysiology, and ecological specialization [127]. New chlorophyte models included *Auxenochlorella protothecoides* (*i*MM627) [128], *Chlorella variabilis* (*i*AJ526) [129], *Chlorella ohadii* (*i*CO1515) [122], *Scenedesmus*/*Tetradesmus obliquus* [130], and the high-lipid *Chromochloris zofingiensis* (*i*CZoF1915) [131] that share a common architectural signature with earlier green algal reconstructions: extensive compartmentalization (typically 8–10 compartments), reaction spaces ranging from ~2000 to >4000 reactions, and a relatively small metabolic gene content (<20% of the genome) supporting a highly interconnected, photosynthetically driven metabolic network. Similar expansions occurred in other lineages. Haptophytes were represented by *Emiliania huxleyi* (iEH410) [123], integrating photophysiology with calcification-associated metabolism; diatoms gained additional reconstructions such as *Thalassiosira pseudonana* (*i*Tps1432) [121] and *Cylindrotheca closterium* (*i*MK1961) [132], extending diatom-specific features like chloroplast–mitochondrial energetic coupling and silicon-associated pathways; eustigmatophytes were expanded by *Nannochloropsis salina* (*i*NS934) [133], notable for its rich glycerolipid metabolism. Most of these GEMs were validated across diverse environmental and trophic scenarios, including autotrophic vs. heterotrophic growth, variable CO_2_ regimes, nitrogen and phosphorus limitation, salinity and light stress, and, in some cases, diel cycles. These validations consistently demonstrated that despite their phylogenetic diversity, eukaryotic algal models converge on similar predictive capabilities: accurate reproduction of growth phenotypes, correct identification of essential pathways under stress, and reliable predictions of carbon and energy redistribution in response to environmental perturbations [134]. Beyond basic validation, the models enabled a wide spectrum of applications, such as identifying bottlenecks in lipid and carotenoid biosynthesis guiding medium design for improved heterotrophic biomass accumulation (*A. protothecoides*) [135], predicting constraints on PUFA production via PKS-like pathways (*S. limacinum*), and exploring silicon-associated energy allocation in diatoms (*T. pseudonana*). Only a subset of models explicitly incorporated omics data as modeling constraints or refinement inputs. *i*NS934 for *Nannochloropsis* used transcriptomics to extend gene–reaction associations and improve lipid pathway coverage and the recently developed *i*Picre1230 for *Picochlorum renovo* integrated RNA-seq datasets from zinc-and salinity-stress experiments to generate context-specific models that revealed dynamic reallocation of photosynthetic and respiratory fluxes, nucleotide recycling strategies, and stress-induced metabolic rewiring. In a more advanced application, *i*MK1961 (*C. closterium*) integrated large-scale environmental meta-omics data to resolve the trophic modes of this diatom across global oceans, including photoautotrophy, heterotrophy, and mixotrophy [132]. Such a framework can be extended to investigate poorly characterized algal species directly within their natural ecological contexts.

In parallel to organism-specific reconstructions, systems biology approaches have begun adopting multistrain modeling frameworks, originally developed for bacteria, to explore metabolic diversity within algal genera [94]. The reconstruction of five *Cyanothece* GEMs (7424, 7425, 7822, 8801, 8802) demonstrated how bacterial multistrain modeling frameworks can be extended toward cyanobacteria with detailed photosynthetic systems. closely related lineages [94]. Strain-specific networks were systematically derived from a curated reference model, enabling direct comparison of diel metabolic programs, nitrogen fixation strategies, and respiratory–photosynthetic partitioning among closely related lineages [94]. Furthermore, Füssy et al., in 2025 generated three independent GEMs for *Phaeocystis* organisms: *P. antarctica*, *P. globosa*, and *P. cordata* [136]. Each metabolic model was reconstructed directly from newly assembled, high-quality genomes with complete compartmentalization and full gene–reaction mapping. *Phaeocystis* metabolic reconstructions were used to interpret lineage-specific physiological strategies by linking predicted flux distributions with metagenomic and transcriptomic evidence from contrasting oceanic provinces [136]. The three GEMs captured differences in nitrogen source utilization, redox balancing, photorespiration, carbon storage, and metabolic pathway usage that mirrored the ecological niches each species occupies, enabling mechanistic explanations for observed biogeographic patterns. Unlike core-model-derived frameworks, the *Phaeocystis* models preserve species-specific metabolic repertoires without collapsing them into a shared reaction set, illustrating how genome-resolved modeling can bridge cellular metabolism with large-scale environmental datasets [136].

Although GEMs provide a comprehensive map of all possible metabolic reactions encoded in an organism’s genome, their unconstrained solution space often contains thousands of mathematically feasible, but biologically unrealistic flux states [137]. Omics-based integration strategies address this limitation by incorporating experimental measurements (transcript levels, protein abundances, metabolite profiles, or fluxomics data) to restrict or weight reaction activity, thereby shifting simulations toward physiologically meaningful solutions [138]. The logic behind these approaches is grounded in the gene–protein–reaction (GPR) associations: when a gene is highly expressed, its associated enzyme is more likely to be active, whereas reactions linked to low- or unexpressed genes are downregulated or removed from the feasible solution space. Depending on the integration method, omics data may (i) adjust reaction bounds (e.g., E-Flux), (ii) penalize flux through reactions with low expression (pFBA-based methods), (iii) identify a minimal reaction set consistent with measured expression patterns (GIMME, iMAT), or (iv) generate multiple condition-specific subnetworks through probabilistic integration (MADE, PRIME) [139]. By reducing the dimensionality of the solution space, omics-constrained models significantly improve the accuracy of growth predictions, essential gene identification, nutrient-uptake behavior, and pathway usage. In algae, where environmental parameters such as light, salinity, nutrient availability, and diel cycles drive complex transcriptional and metabolic responses, omics integration provides a powerful means to connect environmental signals with mechanistic metabolic outcomes [53].

Transcriptomics has become the most widely adopted omics layer for constraining GEMs, largely because RNA-seq offers genome-scale quantitative information on gene expression that can be directly mapped onto GPR associations. Across bacteria, yeasts, and mammalian systems, a broad repertoire of integration methods (e.g., GIMME, iMAT, MADE, E-Flux, PRIME, and context-specific FBA pipelines) have been developed to translate expression levels into reaction constraints, identify condition-specific metabolic states, and predict regulatory shifts under stress or genetic perturbation. These approaches have enabled mechanistic interpretation of transcriptome changes under nutrient limitation, toxin exposure, host–microbe interactions, cancer metabolism, and circadian regulation, illustrating the power of transcriptomics-constrained modeling to capture dynamic metabolic rewiring. Yet, despite the extensive methodological landscape, the application of transcriptomics integration to algal GEMs remains remarkably limited. One of the earliest examples involved *C. reinhardtii*, where transcriptomic datasets were used to update gene–reaction associations, refine light-dependent redox and carbon allocation pathways, and generate condition-specific metabolic predictions that aligned with experimentally measured growth under varying light and nutrient conditions. More recently, a full transcriptomics-constrained reconstruction was implemented for *P. renovo* (*i*Picre1230), where RNA-seq profiles from zinc-stress and salinity-stress experiments were integrated to generate environment-specific metabolic states. These contextualized models revealed stress-dependent remodeling of photosynthetic electron flow, shifts between chloroplast and mitochondrial ATP production, adjustments in nucleotide salvage pathways, and metabolic signatures associated with halotolerance and micronutrient economy. Beyond these isolated cases, transcriptomics integration has been applied more extensively in cyanobacterial modeling, but remains underexplored in eukaryotic algae, representing a major opportunity for future research [140]. Because algae exhibit strong diel regulation, subcellular metabolite partitioning, and pronounced stress-responsive transcriptional plasticity, transcriptomics-constrained GEMs have the potential to greatly improve predictions of environmental adaptation, ecological function, and biotechnological performance across diverse algal lineages [123]. Additional omics tools, specifically metabolomics and proteomics, offer powerful opportunities to further constrain GEMs and reduce the uncertainty inherent to stoichiometric models [54,58]. Metabolomics-guided modeling leverages intracellular or extracellular metabolite abundances to refine reaction reversibility, constrain uptake and secretion rates, infer active metabolic pathways, and benchmark predicted flux distributions [56]. Approaches such as metabolomics-assisted flux balance analysis, dynamic mass-balance modeling, or correlation-based mapping between metabolite fold-changes and flux states have been widely applied in microbial and mammalian systems to uncover redox imbalances, identify thermodynamic bottlenecks, and recover flux predictions consistent with ^13^C-fluxomics profiles. Proteomics integration, although conceptually powerful, remains less explored due to the difficulty of obtaining quantitative enzyme abundances and kinetic parameters [27,54]. Frameworks such as FBA with molecular crowding (FBAwMC), MOMENT [141], GECKO [142], sMOMENT [143], and ETFL [144] incorporate enzyme abundances, molecular weights, or turnover rates (kcat) to bound reaction capacities, dramatically shrinking the solution space and improving the physiological realism of predictions. However, no published algal GEM has yet incorporated quantitative proteomics within enzyme-constrained frameworks such as GECKO or ETFL, and metabolomics datasets are typically used qualitatively rather than as formal model constraints [58]. This contrasts sharply with the situation in bacteria and human studies, where multi-omics pipelines routinely integrate transcriptomics, proteomics, and metabolomics simultaneously to generate highly calibrated, context-specific GEMs [58]. The gap reflects both biological challenges and technical constraints (complex compartmentalization, diverse light-dependent metabolic states, limited enzyme kinetic data for algae, etc.), including incomplete annotation of organelle-specific enzymes and scarce in vivo turnover numbers [57]. As high-resolution MS-based proteomics and metabolomics become increasingly available for algae, these integration frameworks represent a major opportunity for the field: they could enable enzyme-capacity–constrained photosynthetic models, thermodynamically feasible flux reconstructions across diel cycles, precise modeling of nutrient-stress responses, and quantitative prediction of lipid and pigment biosynthesis under industrially relevant conditions [56]. In this sense, multi-omics integration represents the next frontier for elevating algal GEMs toward the predictive accuracy already achieved in microbial and mammalian systems [145].

Multi-omics integration represents the next conceptual and methodological step toward constructing physiologically realistic, data-driven algal metabolic models [75]. Whereas transcriptomics, proteomics, or metabolomics alone refine only one regulatory layer, multi-omics frameworks combine several molecular dimensions simultaneously to generate models that more closely mirror the hierarchical and multilayered control of cellular metabolism [73]. Recent computational advances, including tools such as XomicsToModel [146], rMTA [147], GIMME+pFBA hybrids [148], integrative Bayesian pipelines [149], and big-data-driven workflows [150], enable the systematic combination of heterogeneous omics datasets into stoichiometric models by incorporating reaction confidence scores, refining gene–reaction associations, adjusting flux bounds using enzyme or metabolite constraints, and identifying consensus flux states supported across multiple data layers [73]. Applications in bacteria, fungi, and mammalian systems demonstrate the transformative impact of multi-omics constrained GEMs: bacterial models integrating genomics, transcriptomics, metabolomics, and thermodynamics have revealed previously hidden energy-redox trade-offs; cancer metabolic models combining gene expression, proteomics, and metabolite concentrations successfully predicted tumor-specific vulnerabilities; and fungal reconstructions enriched with transcriptomics–proteomics–metabolomics datasets uncovered regulatory modules governing pathogenicity and nutrient acquisition [75]. In line with the very limited algal works integrating individual omics approaches into GEMs, the application of true multi-omics integration to algal GEMs remains extremely limited [56]. To date, no published algal GEM has yet incorporated proteomics-driven enzyme constraints (e.g., GECKO-like formulations), thermodynamic metabolomics integration, or multi-layer integration of transcriptomics, proteomics, and metabolomics to generate fully context-specific models. Given the extensive algal datasets produced by modern sequencing efforts and marine observatory programs, the absence of broad multi-omics integration represents a major opportunity for the field [74]. Applying these emerging frameworks to algae would enable unprecedented insights into the coordination between photosynthesis, nutrient assimilation, redox regulation, and lipid biosynthesis; disentangle species- and environment-specific metabolic adaptations; and empower the development of predictive, data-rich models capable of explaining and engineering algal behavior across ecological and industrial contexts [56,123]. However, the successful implementation of these next-generation integration strategies fundamentally depends on the availability of high-quality biochemical, genomic, and annotation resources that provide the reaction, gene, metabolite, and pathway information required to support accurate model refinement and analysis [136]. As the algal modeling field prepares to move toward multi-layer, data-driven frameworks, the ecosystem of databases and computational resources becomes increasingly central to enabling model construction, curation, validation, and contextualization [94].

The figure illustrates how different omics layers are used to improve the biological resolution of metabolic models across organisms of increasing complexity. Transcriptomic data can be incorporated using approaches such as GIMME, iMAT, E-Flux, MADE, and PRIME to generate context-specific metabolic models. Proteomic data can be used to constrain enzyme abundance and catalytic capacity through frameworks such as FBAwMC, MOMENT, GECKO, sMOMENT, and ETFL, although such applications remain comparatively limited in algae. Metabolomic data, together with broader multi-omics integration frameworks such as XomicsToModel, rMTA, and Bayesian pipelines, can help link measured metabolite states to predicted metabolic fluxes and pathway activity. The lower panel shows the biological progression of GEMs from bacteria to early eukaryotes and algae, emphasizing increasing metabolic complexity, subcellular compartmentalization, and expanded biotechnological relevance. This progression highlights both the opportunity and the challenge of applying advanced omics-constrained modeling approaches to algal systems, where eukaryotic organization, photosynthetic metabolism, and compartment-specific regulation add additional layers of complexity.

## 4. Data-Driven Modeling in Algae: From Process-Level Prediction to Omics-Aware AI Approaches

**Figure 4:**
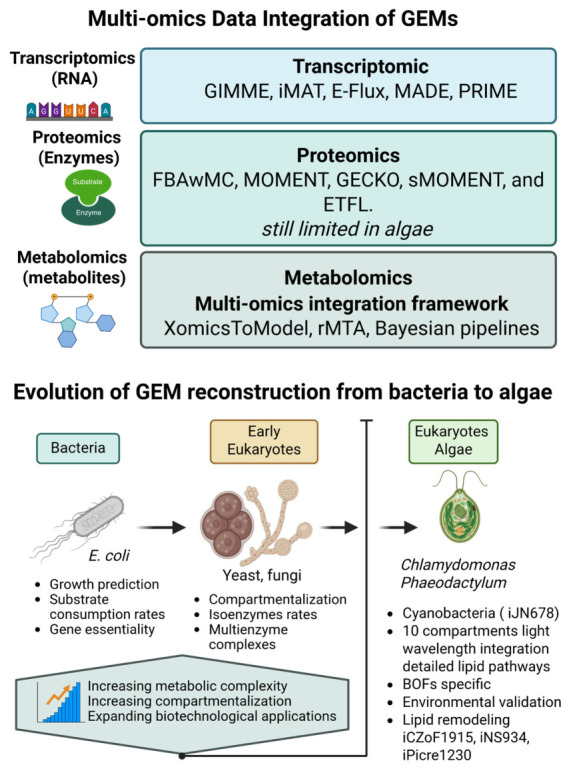
Omics integration across increasing biological complexity in metabolic modeling.

The massive generation of omics and physiological data for different algal organisms under multiple environmental conditions, and specifically, varying a wide range of biotic and abiotic parameters have enabled detailed descriptions, correlations, and even mechanistic explanations of the complex molecular, metabolic, regulatory, and signaling mechanisms employed by algae [151]. GEMs have offered a predictive mechanistic modeling approach and have grown increasingly sophisticated through omics integration and curated biochemical resources (Figure 4) [152,153,154]. However, metabolic models are limited to integrate condition specific datasets to explain the metabolic flux distributions as context-specific models, significantly simplifying the algal biological systems without considering the effect of analyzing or integrating the whole datasets [155,156]. This results in accurate representations of only a reduced layer of the whole biology of algae, capturing the flux profiles represented as metabolic networks, ignoring the encoded regulatory, structural, spatial, or temporal complexity beyond metabolic reactions [157,158]. To overcome the constrained-based metabolic modeling limitations, a complementary class of approaches has emerged: data-driven models, which infer patterns, regulatory programs, and system-level behaviors directly from large-scale datasets without relying on predefined biochemical networks [152,159,160]. Unlike GEMs, which are constrained by known reaction stoichiometries, data-driven methods can exploit the full dimensionality of transcriptomic, proteomic, metabolomic, environmental, and phenotypic datasets to uncover relationships that are nonlinear, multi-layered, or not yet described biochemically [161].

Data-driven modeling approaches allow the prediction of growth, stress responses, metabolic phenotypes, ecological traits, and even bioproduct yields purely from data structure [159,162]. In algae, where extensive environmental and omics datasets continue to accumulate faster than they can be incorporated into mechanistic models [163], data-driven modeling provides a powerful parallel pathway to analyze complexity, generate new hypotheses, and reveal biological mechanisms that may later be formalized within GEMs [160]. This data-driven modeling comprehends a continuum of increasingly computational approaches that evolved in response to growing data volume, dimensionality, and complexity [159].

Early statistical learning methods (e.g., regression [164], clustering [165], principal component analysis [166], and correlation-based network inference [167]) have been extensively used to detect dominant trends, variance structure, and co-regulated modules in algal transcriptomes, metabolomes, and environmental datasets [168]. As omics datasets became larger and more mathematically complex, statistical learning methods evolved into ML, which introduced formal training paradigms and predictive modeling [159]. ML algorithms are designed to learn directly from data, identifying relationships and interactions that are often imperceptible. ML approaches can be broadly classified into supervised methods, which learn relationships between inputs (e.g., nutrient levels, gene expression) and labeled outputs (e.g., growth rate, lipid content) [159]. For instance, Linear and Logistic Regression models have been employed to predict continuous growth phenotypes and binary classification tasks assuming linear relationships [169]. Meanwhile, unsupervised methods uncover hidden groupings or latent structures without prior labels [169].

**Figure 5:**
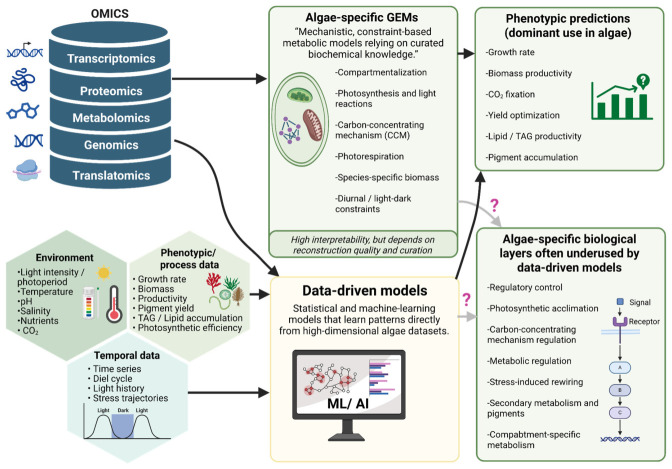
Data-driven modeling in algae: current applications and underexploited potential.

Unsupervised ML is particularly useful for identifying stress-responsive metabolic states or community-level signatures in algal systems. Further advances in data-driven modeling led to the widespread adoption of deep learning (DL) methods, which are built upon artificial neural networks inspired by the hierarchical organization of biological information processing [161,170]. In DL models, interconnected layers of neurons progressively transform input data into increasingly abstract representations, allowing complex nonlinear relationships to be learned directly from high-dimensional inputs [170]. Common DL architectures include feedforward neural networks for phenotype prediction, convolutional neural networks (CNNs) for extracting spatial patterns from images or structured matrices, recurrent neural networks (RNNs) and long short-term memory (LSTM) models for capturing temporal dependencies such as diel expression cycles, and autoencoders for dimensionality reduction and latent feature discovery in multi-omics datasets [161]. More recently, graph neural networks (GNNs) have emerged as powerful tools for learning from biological networks, offering a natural framework to represent metabolic, regulatory, or interaction graphs [171]. In the context of algal research, data-driven methods have been predominantly applied to optimize cultivation conditions, predict growth or biomass accumulation, estimate lipid or pigment yields, and evaluate environmental performance in engineered or wastewater treatment systems [171,172,173]. The direct use of ML and AI to extract biological insight from algal transcriptomic, proteomic, metabolomic, or integrated multi-omics datasets remains limited to a small number of exploratory studies. As a result, current ML/AI applications in algae largely operate at the phenotype or process level, rather than being leveraged as discovery tools for molecular regulation (Figure 5) [173].

The current ML and AI research in algae can be understood primarily as an effort to model, predict, and optimize physiological performance under complex and often poorly constrained cultivation environments. Rather than interrogating molecular regulation directly, these approaches treat algal cells as dynamic input–output systems, where multivariate environmental and operational parameters are mapped to macroscopic phenotypes such as growth rate, biomass productivity, carbon fixation, or metabolite yield [174]. Within this framing, ML serves as a flexible surrogate for mechanistic growth models, enabling the capture of nonlinear interactions among cultivation variables and supporting decision-making in experimental design, process optimization, and operational control [173,175]. A representative example is the use of ML pipelines to learn nonlinear response surfaces for biomass productivity and CO_2_ biofixation from relatively small but structured experimental designs. In *C. vulgaris*, Hossain et al. trained multiple supervised regressors such as boosted regression trees (BRT), artificial neural networks (ANN), and support vector regression (SVR) and then coupled each learner to Bayesian optimization (BOA) for automated hyperparameter tuning and k-fold validation, enabling joint prediction of biomass productivity and CO_2_ fixation while reducing the need of multiple experiment designs [171]. The authors further combined the best-performing predictor with a metaheuristic (crow search algorithm) to compute global optima for cultivation variables (temperature, light–dark cycle, and N:P ratio), explicitly positioning ML as a decision-support layer for multi-objective process optimization. Ultimately, BOA strategies were also used for downstream process endpoints such as biodiesel yield, where BOA-tuned ANN and SVR models were benchmarked against prior statistical models and validated with additional literature data, again emphasizing rapid yield estimation and reduction in laboratory burden rather than mechanistic interpretation [176].

A second major category of applications focuses on empirical growth modeling and variable prioritization under operationally realistic, and often highly variable cultivation conditions, especially when the system is driven by fluctuating outdoor conditions and multi-constraint cultivation practices. Mazzelli et al. implemented multivariate projection methods (PCA) and Partial Least Squares regression (PLS) to compress one-year outdoor photobioreactor monitoring and build a usable empirical predictor for growth rate and productivity; PCA separated major sources of variance into interpretable components associated with environmental versus cultivation conditions, while PLS achieved strong predictive performance for specific growth rate and productivity, with an explicit motivation of producing a model that end users can calibrate and deploy more easily than heavily parameterized mechanistic formulations [172]. In parallel, ANN-based frameworks are frequently used to learn nonlinear growth dynamics across heterogeneous datasets and then extract ranked drivers via sensitivity/feature-importance analyses. For example, Liyanaarachchi et al. developed a multilayer perceptron ANN (23–20–1) for *C. vulgaris* growth prediction using a broad set of process inputs (irradiance, photoperiod, temperature, aeration rate, CO_2_ fraction, inoculum density, cultivation time, plus nutrient concentrations), achieving high agreement with experiments and explicitly quantifying variable importance (CO_2_ supply as the dominant factor within their tested range; nitrogen and copper among the strongest nutrient effects) [177]. Under these approaches, ML methods compress large monitoring datasets into a small number of latent components associated with environmental forcing versus controllable operational parameters, allowing robust prediction of growth rate and productivity while maintaining interpretability and ease of deployment. Furthermore, artificial neural network models trained on heterogeneous experimental datasets are widely used to predict algal growth across broad parameter spaces [174,177]. Critical model parameters considered and incorporated are irradiance, photoperiod, temperature, aeration rate, CO_2_ concentration, cultivation time, inoculum density, and nutrient composition as inputs, achieving high predictive accuracy even when interactions among variables are strongly nonlinear. ML strategies have allowed the trained models to rank the relative influence of individual parameters and thereby inform practical cultivation strategies [178].

More recent studies extend this process-level paradigm with time-series DL and control-oriented applications that explicitly encode history dependence (e.g., light acclimation) and support decisions about harvesting/operation [173]. Yeh et al. used 50-day outdoor cultivation data for the diatom *P. tricornutum* to compare traditional growth models (e.g., Monod/Haldane-type forms) against SVR and an LSTM recurrent neural network; the ML models outperformed traditional models because they incorporated light history as input, with the LSTM specifically capturing acclimation-driven nonlinearities and enabling downstream applications such as a biomass “soft sensor” and an optimal harvest strategy [173]. At a larger techno-operational scale, Long et al. used ML to predict light distribution patterns under mutual shading and then link predicted light availability to growth-rate prediction, using these coupled models to design semi-continuous cultivation regimes that maintain high productivity; the approach is positioned as a practical route to operationalize complex light–growth couplings where mechanistic optics/biokinetics models are difficult to generalize [175]. Similar strategies have been developed in wastewater treatment contexts, where supervised regression models are trained on physicochemical descriptors such as nutrient load, chemical oxygen demand, pH, and hydraulic retention time to predict biomass yield or nutrient removal efficiency [179]. In these studies, multiple algorithms are typically benchmarked and then embedded within evolutionary or genetic optimization routines to identify operational regimes that maximize performance, while feature-importance and sensitivity analyses are used to rationalize which environmental drivers most strongly influence algal growth [180].

To date, the majority of ML- and AI-based approaches in algal research have focused on optimizing growth, biomass productivity, or system-level performance [181]. However, these objectives represent only a subset of the biotechnological potential encoded within algal biology (metabolism, regulation, signaling, etc.). In many industrial and environmental contexts, the primary goal is not maximal biomass accumulation, but rather the selective enhancement of specific high-value compounds, such as pigments, lipids, antioxidants, or specialty metabolites, whose synthesis are part of secondary metabolic pathways and decoupled from growth and tightly regulated [182,183]. The production of high-value compounds introduces additional complexity, as compound accumulation frequently emerges from nonlinear trade-offs between growth, stress response, and metabolic rewiring. Therefore, data-driven models have begun to be explored not only as predictors of algal performance, but also as tools to navigate and optimize compound-specific metabolic outputs under multi-parameter cultivation conditions [175,182]. In this context, data-driven models (mainly supervised approaches) are typically trained on multivariate cultivation datasets incorporating environmental parameters such as light intensity and photoperiod, temperature, nutrient concentrations (e.g., nitrogen, phosphorus, trace metals), salinity, CO_2_ availability, and stress-inducing conditions known to trigger secondary metabolism [177,182]. A range of ML and DL models, including artificial neural networks, support vector regression, random forests, gradient-boosting methods, and hybrid ML–optimization frameworks, have been employed to capture nonlinear interactions among these variables and to identify optimal operating regimes [171,176,177]. Such approaches have been successfully applied to enhance carotenoid production, including fucoxanthin in *Isochrysis galbana* and *P. tricornutum*, as well as astaxanthin in *H. pluvialis*, where ML-guided strategies outperform empirical optimization [184].

Beyond yield optimization, recent studies increasingly emphasize non-invasive monitoring and real-time assessment of metabolite production. DL approaches that combine computer vision, hyperspectral or fluorescence spectroscopy, and convolutional neural networks have been used to directly infer intracellular pigment concentrations from optical signals, enabling rapid, high-throughput, and potentially real-time quantification without destructive sampling [184,185]. Similar data-driven strategies have been extended to cyanobacteria and wastewater-based algal biorefineries, where ML models are trained to simultaneously predict biomass, pigment accumulation, lipid content, or nutrient removal efficiency, often within multi-objective optimization frameworks that balance productivity, resource utilization, and environmental performance [171,179]. However, most ML/DL applications in algae remain empirically driven and operate at the phenotype or process level, relying on external physicochemical or optical descriptors rather than directly integrating intracellular omics data to resolve regulatory or pathway-level control of secondary metabolism. This contrasts with more mature applications in bacteria, fungi, plants, and mammalian systems, where ML and AI are increasingly integrated with omics data to infer biosynthetic gene clusters, regulatory circuits, enzyme activity, and pathway-level bottlenecks underlying high-value compounds production.

GEMs provide mechanistic predictions based on curated biochemical networks and integrated multi-omics datasets, whereas data-driven approaches, including statistical learning, machine learning, and AI, infer patterns directly from high-dimensional environmental, phenotypic/process, and temporal datasets. In algae, both approaches have been used predominantly to predict phenotypic and process-level outcomes, such as growth rate, biomass productivity, CO_2_ fixation, lipid/TAG accumulation, pigment yield, and cultivation performance under defined light and nutrient regimes. However, their potential to interrogate deeper algae-specific biological layers, including regulatory control, photosynthetic acclimation, carbon-concentrating mechanism regulation, signaling pathways, metabolic rewiring, stress responses, and secondary metabolism, remains largely underexplored.

## 5. Toward Predictive Algal Systems Biology: Context-Specific GEMs, Expanded Constraints, and Hybrid ML–GEM Frameworks

Algal research has benefited from a massive expansion of genomic, transcriptomic, proteomic, metabolomic, and physiological datasets spanning diverse taxa, environmental regimes, and cultivation strategies [3,168]. The generation of omics data in algal organisms has enabled the reconstruction of increasingly curated GEMs and the development of context-specific simulations that capture condition-dependent metabolic flux distributions [151]. Expanding algal GEM collection and applications have provided valuable insight into central carbon metabolism, nutrient assimilation, photosynthetic energy conversion, and stress-responsive metabolic reprogramming across different algal lineages and growth conditions [186]. In parallel, data-driven approaches have used physiological and environmental measurements to predict growth, optimize cultivation conditions, and monitor selected bioproducts, particularly in controlled or engineered systems [173].

In bacteria, fungi, plants, and mammalian cells, comparable increases in data availability have driven a broader methodological evolution in computational modeling [22,157]. Omics datasets are now used not only to contextualize metabolic fluxes, but also to constrain gene expression, enzyme allocation, resource investment, and thermodynamic feasibility within mechanistic frameworks [155,187]. These advances have shifted modeling from generic GEMs toward context-specific and expanded formulations that capture biological layers beyond metabolism, including transcriptional and translational costs, protein turnover, condition-dependent proteome allocation, and resource trade-offs [188,189]. In bacteria and fungi, such models have revealed condition-dependent pathway activation, metabolic strategies under nutrient limitation or stress, and trade-offs between growth, maintenance, and secondary metabolism [190]. In plants, tissue- and environment-resolved models have provided insight into organ-specific resource allocation, diel metabolic partitioning, and abiotic stress responses, while related approaches in mammalian systems have been applied to developmental, disease, and differentiation-associated metabolic reprogramming [191,192].

In algae, however, omics integration into GEMs remains comparatively limited in both scope and frequency. As discussed in Section 3, only a small number of studies have incorporated transcriptomic data into algal GEMs, and these efforts have largely demonstrated feasibility rather than systematically interrogating condition-dependent metabolic regulation [90]. Consequently, many algal GEMs are still used as generic representations of metabolic potential, with relatively few context-specific models designed to examine metabolic rewiring across environmental gradients, stress conditions, ecological niches, or bioprocess regimes [193]. This limits the ability to use existing algal datasets to address how metabolism reorganizes in response to light availability, nutrient limitation, salinity, temperature, metal stress, or biotic interactions.

Expanding context-specific algal modeling is therefore a critical intermediate step toward more advanced integrative frameworks. Such models could clarify how light–dark cycles and fluctuating irradiance reshape metabolism, how nutrient limitation redirects carbon toward growth, storage compounds, or secondary metabolites, and how redox and energy-balancing constraints vary across taxa and ecological niches. They could also be used to compare metabolic adaptations to salinity, temperature, or metal stress, distinguish free-living and symbiotic lifestyles, and identify conserved versus lineage-specific modes of metabolic regulation [194]. Importantly, context-specific models would transform transcriptomic, proteomic, and metabolomic signatures from descriptive lists of differentially expressed genes or metabolites into system-level explanations of pathway usage, energetic trade-offs, redox balancing, resource allocation, and environmental adaptation.

Despite their value, conventional GEMs and context-specific models remain constrained by simplified representations of cellular biology. Traditional GEMs encode metabolism through gene–protein–reaction associations and fixed stoichiometric coefficients, then optimize flux distributions toward a static biomass objective that assumes constant cellular composition and resource requirements [195]. Although transcriptomic or proteomic data can be used to activate, deactivate, or bound reactions, these approaches do not explicitly represent the molecular processes that determine enzyme abundance, catalytic capacity, macromolecular investment, or resource competition. As a result, biomass composition, maintenance costs, and cellular resource allocation are often treated as fixed, even though these properties are highly dynamic and strongly influenced by environment, growth rate, and stress state [196].

**Figure 6:**
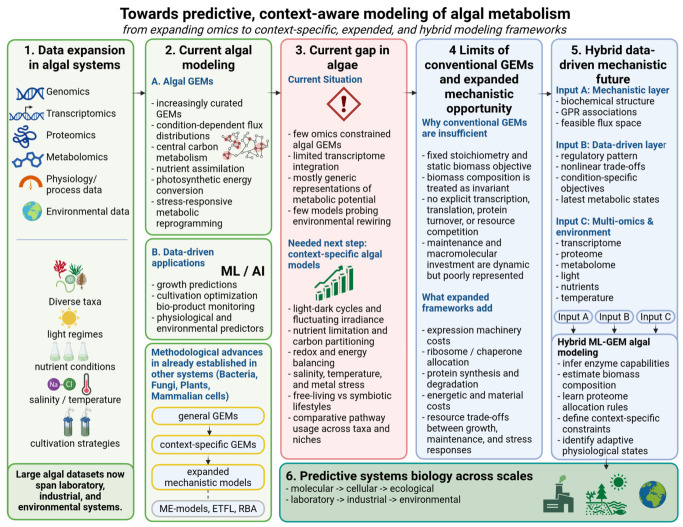
Toward predictive, context-aware modeling of algal metabolism through hybrid mechanistic and data-driven frameworks.

Addressing these limitations requires modeling biological layers beyond metabolism, including transcription, translation, protein synthesis and degradation, ribosome and chaperone allocation, and the energetic and material costs of maintaining the expression machinery. These processes directly constrain metabolic capacity by limiting enzyme availability and imposing trade-offs between growth, maintenance, and stress responses that stoichiometry-based models alone cannot capture. In algae, additional complexity arises from photosynthesis, compartmentalized gene expression, diel regulation, and interactions with microbial communities in the phycosphere, where nutrient, vitamin, and signaling exchange can modulate metabolism. While expanded frameworks such as ME-models, ETFL, and resource balance analysis have been developed most successfully in prokaryotic systems, extending similar approaches to eukaryotic photosynthetic organisms remains more demanding because of compartmentalization, regulatory complexity, and dynamic allocation across cellular subsystems (Figure 6) [144,187,197].

Data-driven modeling can help overcome these limitations and complement both classical and expanded GEM frameworks [23,159]. Unlike constraint-based models, which require predefined objective functions, kinetic parameters, and explicit molecular mechanisms, machine learning and AI approaches can infer latent structure, regulatory priorities, and nonlinear trade-offs directly from high-dimensional omics and environmental datasets [161]. This is particularly relevant for algae, where many physiological states are not governed primarily by growth maximization, but by photoprotection, redox balancing, nutrient scavenging, storage compound accumulation, or stress adaptation. Data-driven models can therefore help infer condition-specific regulatory programs, metabolic states, enzyme capacities, biomass composition, proteome allocation rules, and alternative objective functions that are difficult to encode explicitly in current ME-, ETFL-, or RBA-based formulations.

Hybrid data-driven mechanistic modeling offers a practical path forward for algal systems. In such frameworks, GEMs define the feasible biochemical space, while machine learning models learn relationships between omics-derived cellular states, environmental inputs, and metabolic phenotypes within that constrained space [24]. For example, machine learning models trained on GEM-derived flux distributions constrained by transcriptomic and environmental data can learn nonlinear relationships between gene expression, environmental conditions, and system-level metabolic outputs [198]. This hybrid strategy addresses limitations of each approach: machine learning can capture regulatory effects, nonlinear dependencies, and condition-specific trade-offs that are difficult to encode mechanistically, while GEM-derived constraints keep predictions anchored to known biochemical structure and reduce physiologically implausible solutions [157].

These advances point toward a convergent modeling paradigm for algae in which mechanistic and data-driven approaches operate as complementary, iterative components. GEMs provide biochemical structure, gene–protein–reaction associations, and feasible flux spaces; expanded formulations add expression, resource allocation, and energetic costs; and data-driven models provide a flexible inference layer for regulatory structure, condition-specific objectives, nonlinear trade-offs, and adaptive physiological states [159,170,189]. For algae, whose biology is shaped by dynamic light environments, compartmentalized metabolism, ecological coupling, and extensive metabolic plasticity, such hybrid frameworks offer a scalable route to predictive, context-aware modeling across laboratory, industrial, and environmental settings (Figure 6).

Algal systems occupy a unique position at the intersection of photosynthesis, metabolism, and environmental adaptation, yet their computational modeling remains less mature than that of bacterial, fungal, plant, and mammalian systems despite rapid omics expansion [196]. Moving beyond growth-centric models toward integrative frameworks that capture regulatory plasticity, metabolic rewiring, resource allocation, and ecological function will be essential for the next stage of algal systems biology. As datasets continue to grow in scale and resolution, the integration of context-specific GEMs, expanded mechanistic models, and data-driven inference can transform algal research from descriptive analysis toward predictive systems biology, with broad relevance for biotechnology, environmental monitoring, and ecosystem-level modeling [3,164].

The figure summarizes how expanding algal genomic, transcriptomic, proteomic, metabolomic, physiological, and environmental datasets can support the transition from generic GEMs to context-specific, expanded, and hybrid ML–GEM frameworks. Current algal models predict flux distributions, growth, nutrient assimilation, photosynthetic energy conversion, and bioproduct-related traits, but relatively few models integrate omics constraints or probe condition-dependent metabolic rewiring. Context-specific algal modeling can address light–dark cycles, nutrient limitation, carbon partitioning, redox and energy balancing, salinity, temperature, metal stress, and lineage- or niche-specific pathway usage. Expanded frameworks such as ME-models, ETFL, and RBA can incorporate expression costs, protein allocation, maintenance demands, and resource trade-offs, while hybrid ML–GEM approaches combine biochemical constraints with data-driven inference to learn nonlinear regulatory patterns, context-specific objectives, enzyme capacities, biomass composition, and adaptive physiological states across laboratory, industrial, and environmental scales.

## Figures and Tables

**Table 1:** Algal-specific metabolic regulation and carbon-allocation strategies revealed by omics and constraint-based modeling.

Species	Culture Conditions	Growth/Biomass Response	Metabolite or Product Response	Regulatory/Metabolic Mechanisms Identified
*Nannochloropsis gaditana*	Nitrogen starvation experiment: F/2 medium; 23 ± 1 °C; continuous light at 100 µmol photons m^−2^ s^−1^; pre-culture for 4.5 d in N-rich F/2 containing 0.075 g/L NaNO_3_, then transferred to either N-replete F/2 or N-free medium for 7 d. Sequential batch reactor: f/2 medium, 12 h/12 h light/dark cycles; comparable starting nitrate concentrations 78.5–84.2 mg NO_3_/L.	Under N-replete vs. N-starved conditions, dry biomass after 5 d was 0.43 ± 0.03 g/L and 0.46 ± 0.05 g/L, respectively. In SBR culture, maximum specific growth rate was 0.23 ± 0.04 gVSS/gVSS·d using VSS and 0.16 ± 0.08 gVSS/gVSS·d using the OD_550_–VSS correlation.	TAG increased from 70 to 380 µg/mg dry weight, reaching 38% of total biomass under N starvation. Polar glycerolipids decreased from 60 to 20 µg/mg dry weight. MGDG decreased by about two-thirds. FFA content almost doubled.	N starvation induced de novo fatty acid synthesis rather than simple membrane lipid recycling. TAGs carried signatures of newly synthesized FA, while a smaller fraction originated from polar glycerolipid turnover. N-starved cells decreased chloroplast galactolipids but maintained enough thylakoid integrity for photosynthesis. PSII, PSI, and cytochrome b_6_f decreased; PSII was most affected, reducing linear electron flow. Cyclic electron flow was activated to compensate. In SBRs, nitrate uptake was decoupled from biomass growth; maximum nitrate uptake was 210 ± 11.2 mg NO_3_/gVSS·d, supporting luxury uptake/internal N storage.
*Phaeodactylum tricornutum*	Fucoxanthin productivity experiment: CCAP 1055/1; F/2, F/2 + nitrate, and F/2 + nitrate + phosphate media; inoculum 1 × 10^6^ cells/mL; 22 °C; 250 µmol m^−2^ s^−1^ white LED; 16:8 h light/dark; 15 replicates per assay. F/2 and F/2 + N sampled on days 3, 5, 7, 9; F/2 + N + P sampled through day 20. Ocean acidification/light experiment: ambient CO_2_ 390 ppmv vs. elevated CO_2_ 1000 ppmv; light levels 60, 200, 460 µmol m^−2^ s^−1^. Modeling: iLB1027_lipid GEM constrained with FTIR-derived biomass composition under N-replete to N-starved growth.	In F/2 + N + P, cell concentration reached 30 million cells/mL by day 20. In F/2 + N, cell concentration plateaued at ~15 million cells/mL. In F/2 alone, cell concentration reached ~10 million cells/mL. Elevated CO_2_ increased specific growth rate by ~12–18% relative to ambient CO_2_, with highest growth at medium light.	Fucoxanthin reached ~17 ppm in F/2 + N + P. In F/2 + N, fucoxanthin plateaued at ~3 ppm. In F/2 alone, fucoxanthin remained near ~1.2 ppm. P. tricornutum is also reported as a chassis for EPA, fucoxanthin, neutral lipids/TAG, chrysolaminarin, recombinant proteins, triterpenoids, and PHB.	N and P co-availability controlled nitrate assimilation, photosynthetic activity, cell density, and fucoxanthin production. In F/2 + N, nitrate remained high (~11–9 mM until day 9), but phosphate depletion limited growth and fucoxanthin accumulation. In F/2 + N + P, 80% of nitrate was consumed by day 9, showing that nitrate use depended on phosphate availability. Low light tends to favor fucoxanthin/EPA, whereas high light and N limitation favor storage compounds such as chrysolaminarin and TAG. Elevated CO_2_ downregulated carbon-concentrating mechanisms in a light-dependent manner, saving energy for growth. The iLB1027_lipid model includes 1027 genes, 4456 reactions, and 2172 metabolites across six compartments and predicts carbon partitioning into storage lipids/carbohydrates depending on nutrient and light/dark state.
*Picochlorum renovo*	High-productivity study: simulated outdoor diel light/temperature cycling; screened from >300-strain collection; saline cultivation. Formate/photoformatotrophy: chloroplast expression of formate dehydrogenase; pH 6 screening; formate at 5, 10, 25 mM; high CO_2_ 2% and ambient CO_2_ 0.04% conditions. Protein secretion: genetically engineered strains secreting mCherry using native signal peptides.	Diel biomass productivity: 34.3 g m^−2^ day^−1^ from h 6 to h 30. Dark biomass loss: 0.25 g m^−2^ h^−1^ during first 11 h dark period and 0.46 g m^−2^ h^−1^ in second dark period. Peak growth at 35 °C; growth capacity up to 40 °C. Growth at salinity up to 107.5 g/L. Stationary-phase composition at 30 h: 10% FAME, 20% protein, 59.5% carbohydrates, 10.5% unidentified biomass AFDW. In FDH strains, ambient CO_2_ + formate allowed growth beyond WT stationary phase, with final OD > 4.3× WT.	FAME content varied from 8.5–16.2% AFDW, with C16:0, C16:3, C18:1n9, C18:2n6, and C18:3n3 as major lipid fractions. Glucose from biomass hydrolysis decreased from 52% to 1.4% AFDW after inoculation into fresh medium. FDH strains used 100% of 5 mM formate and 78 ± 1% of 10 mM formate; ^13C-formate incorporation into biomass was 6.0% and 8.6% for 5 and 10 mM formate. Protein secretion platform: mCherry secretion titer 0.37 mg/L, productivity 0.19 mg/L/day, and ~24% of mCherry secreted.	High productivity is associated with halotolerance, thermotolerance, diel growth, and dynamic carbohydrate/protein allocation. High-salinity transcriptomics identified differential regulation of proline metabolism and candidate haloresponsive genes, including polyketide synthases, inositol phosphorylceramide glucuronosyltransferase, ceramide kinase, and DNA repair/recombination genes. FDH expression linked formate oxidation to chloroplast redox metabolism, reducing formate toxicity and enabling formate-derived carbon assimilation. Protein secretion used native N-terminal signal peptides predicted by SignalP/DeepLoc, enabling extracellular product recovery.
*Haematococcus pluvialis*	UTEX 2505; 1 L glass bottle PBRs, 0.5 L working volume, atmospheric air at 1.5 L/min, cool white LED, batch mode 26 d, 28 ± 2 °C. CCD variables: light intensity 70–270 µmol m^−2^ s^−1^, initial nitrate 0–164.8 mg NO_3_^−^-N/L, initial phosphate 0–212.67 mg PO_4_^3−^-P/L, initial biomass 0.10–0.90 g/L. Seed culture: BBM, 85 µmol m^−2^ s^−1^, 28 ± 2 °C.	ANN optimization forecasted optimal biomass concentration 1.02 g/L on day 13. Red-stage optimized condition experimentally gave biomass concentration 0.94 g/L after 18 d. Additional growth details 1.53 g/L biomass in 15 d for SAG 34–1b under 22 °C, 250 mg/L sodium bicarbonate, 150 mg/L N, and 40 mg/L P.	Optimized astaxanthin concentration: 28.76 mg/L, equivalent to 3.06% DW, on day 18 under nitrate-free conditions. Astaxanthin productivity: 1.60 mg/L/day. Astaxanthin contents of 3.01%, ~1.9%, 3.8%, 3.2%, 2.4% without Fe(II), and 3.9% under different stress/light/CO_2_ configurations.	Astaxanthin production is a two-phase process: green stage favors biomass; red/stress stage favors secondary carotenoid accumulation. Nitrogen availability supports proteins, chlorophyll, energy transfer, and DNA; nitrogen deficiency suppresses cell proliferation but promotes non-nitrogenous compounds, including neutral lipids and carotenoids. Biomass was maximized at intermediate nitrate/phosphate; astaxanthin was maximized in the absence of nitrate with sufficient phosphate and high light.
*Chlorella vulgaris*	Wastewater study experiments: strain FACHB31; maintained in BG11; domestic sewage from Guodian North Sewage Treatment Plant; wastewater CODcr 195–200 mg/L, NH_3_-N 42–46 mg/L, TP 4.1–4.3 mg/L, TN 81–86 mg/L, pH 8.1–8.2. BG11 pre-culture at 50 µmol m^−2^ s^−1^, 24 h light, 25 °C. GEM study: strain UTEX 395; autotrophic, heterotrophic, and mixotrophic conditions; model-guided medium supplementation. Non-axenic PBR study: tap-water-based media, flat-plate PBRs with no mixer/plain mixer/complex mixer.	Wastewater study: highest specific growth rate 0.1675 d^−1^; highest biomass productivity 0.0229 g/L/day. Biomass calibration: Nx = 0.3638 OD_680_ − 0.0294, R^2^ = 0.9983. Non-axenic PBR study: maximum biomass concentration 1.0 g/L and maximum specific growth rate 2.0 d^−1^. GEM-guided study: addition of tryptophan and methionine increased growth relative to base medium.	Wastewater nutrient-removal outputs: TN 94.01%, TP 90.08%, NH_3_-N 97.33%, COD 85.37%. C. vulgaris can accumulate lipids up to 60% dry weight. Model analysis found changes in carbohydrates, proteins, lipids, and RNA across trophic regimes.	Growth in wastewater is coupled to N/P/COD assimilation, but logistic models still miss nutrient removal, aging, and cell death effects. GEM iCZ843 predicts nitrogen starvation redirects metabolism toward storage compounds, including lipids and carbohydrates. Heterotrophic growth increased TAG, PG, PI, and PE; photoautotrophic growth increased MGDG, consistent with photosynthetic membrane demand. Mixotrophic growth can be approximated as the sum of heterotrophic and photoautotrophic growth rates.
*Chlamydomonas reinhardtii*	High CO_2_/flue gas study: photon flux 200 µmol photons m^−2^ s^−1^, 24 h light, ~23.8 °C; pure CO_2_ at 1.2 ± 0.4%, 6.8 ± 1.0%, 17.1 ± 2.4%; flue gas at 17.4% CO_2_/102 ppm NOx, diluted flue gas 6.3% CO_2_/36 ppm NOx and 10.0% CO_2_/58 ppm NOx. Trophic acclimation: CC-124/137c; TAP mixotrophic medium with 17.5 mM acetate, TM autotrophic medium; 42 µmol m^−2^ s^−1^, constant light; ~1-year acclimation to autotrophy or mixotrophy; initial densities 2 × 10^4^ cells/mL for mixotrophic and 3 × 10^4^ cells/mL for autotrophic cultures. Metabolic modeling: photobioreactor medium designed to reach 4.5 g/L dry weight.	Pure CO_2_ experiment: biomass after 5 d was 1.00 ± 0.03 g/L at 1.2% CO_2_, 1.17 ± 0.05 g/L at 6.8% CO_2_, and 0.68 ± 0.15 g/L at 17.1% CO_2_. Cell diameter: 6.6 ± 0.8 µm, 7.4 ± 0.1 µm, and 5.7 ± 0.9 µm, respectively. Dry weight per cell: 1184 ± 21, 1215 ± 35, and 1258 ± 63 pg/cell. Flue gas: biomass 1.11 ± 0.07 g/L under undiluted flue gas vs. 1.87 ± 0.03 g/L under 4.8% pure CO_2_ after 4 d. Chemostat modeling: biomass yield on light 1.25 g/mol photons; ATP biomass-formation requirement 109 mmol/g; ATP maintenance 2.85 mmol/g/h.	High-value products discussed include hydrogen under sulfur starvation/anaerobiosis, starch, lipids/TAG, fatty acids, pigments, terpenoids, recombinant proteins, and bioactive compounds. Fatty acid desaturase study: ω-3 PUFA account for >50% of total fatty acids; crfad7 mutant showed >65% reduction in total ω-3 fatty acids.	High CO_2_ above the optimum suppressed growth; inhibition was attributed to high CO_2_ itself rather than NOx/SO_2_ pollutants or low O_2_. Trophic acclimation changed primary metabolism: mixotrophic acclimation increased carboxylates and amino acids; autotrophic acclimation increased sugars; free fatty acids and acylglycerols showed the most stable dynamics. The prior trophic regime produced metabolic legacy effects after switching conditions, consistent with long-term acclimation, possible epigenetic regulation, mutation accumulation, and rewiring of carbon/energy allocation. FAD7 localizes to the chloroplast but affects both plastidic and extraplastidic lipids, indicating acyl trafficking or lipid exchange between compartments.
*Isochrysis galbana*	Marine microalga; seawater salinity 27 ± 1, pH 8 ± 0.5; 23 ± 2 °C; light intensity 2000 lx; photoperiod 16 h dark/8 h light; 30 d culture; stock in Conway’s medium for 14 d; inoculum adjusted to 2.5 mg/mL wet biomass; 150 mL medium-enriched seawater; phytohormone supplementation.	Growth monitored by OD_680_ every alternate day; fresh weight and dry weight measured.	Fucoxanthin yield predicted using ML; Random Forest achieved R^2^ 0.809, RMSE 0.776 without hormone descriptors and R^2^ 0.839 with hormone descriptors. Methyl jasmonate 0.2 mg/L was identified as an effective phytohormone.	Phytohormones modulate fucoxanthin production. ML captured nonlinear relationships between hormone treatments, growth, morphology, biomass, and fucoxanthin yield. Fucoxanthin functions in photon absorption, photosynthesis regulation, and chlorophyll photoprotection. Absence of a cell wall facilitates fucoxanthin extraction.

**Table 2:** Genus-specific metabolic regulation revealed by omics integration and constrained-based modeling in representative algae.

Genus/Representative Species	Biological Context	Experimental/In Silico Validations	Regulatory and Metabolic Mechanisms Identified	Mechanistic Output or New Insight
*Nannochloropsis* spp./*N. salina*, *N. gaditana*	Nitrogen limitation, lipid accumulation, EPA/TAG production, and light/CO_2_-dependent metabolism	Genome-scale metabolic modeling, transcriptome-supported reconstruction, lipidomics, photosynthetic physiology, and nitrogen-source simulations	Nitrogen starvation promotes carbon redistribution toward fatty acid and TAG biosynthesis while reducing investment in chloroplast membrane expansion. Lipidomic analyses indicate that TAG accumulation is largely supported by de novo fatty acid synthesis, with a smaller contribution from turnover of polar glycerolipids. Photosynthetic remodeling includes reduction in PSII, PSI, and cytochrome b_6_f components, with increased cyclic electron flow compensating for reduced linear electron transport. The iNS934 reconstruction explicitly represents nitrogen assimilation, PUFA biosynthesis, EPA formation, and TAG-related reactions.	*Nannochloropsis* represents a lipid-specialized regulatory strategy in which nitrogen limitation decouples biomass production from carbon fixation and channels carbon/reductant toward neutral lipid storage. The iNS934 model enabled prediction of growth across media conditions and proposed 82 knockout strategies for TAG improvement, linking omics-supported reconstruction to strain-design hypotheses.
*Phaeodactylum*/*P. tricornutum*	Diatom carbon partitioning under nitrogen status, diel metabolism, and lipid/EPA/fucoxanthin bioproduction	Genome-scale metabolic modeling, FTIR-derived biomass composition, nitrogen-stress physiology, transcriptomics, reverse genetics, and enzyme perturbation studies	The iLB1027_lipid reconstruction integrates compartmentalized diatom metabolism with experimentally measured lipid, carbohydrate, and protein composition. Flux simulations indicate that fixed carbon during the light phase can be partitioned into chrysolaminarin, neutral lipids, or biomass depending on nitrogen and diel state. Nitrogen stress remodels intermediate metabolism, including carbon flow through TCA-associated reactions and nitrogen assimilation. Enzyme-level studies identify regulatory nodes such as nitrate reductase, phosphoenolpyruvate carboxykinase, DGAT, AGPAT, malic enzyme, and methylcrotonyl-CoA carboxylase as control points affecting lipid or TAG accumulation.	Diatom metabolism cannot be accurately represented with a single static biomass objective. *Phaeodactylum* requires condition-specific biomass composition and compartment-aware flux analysis to capture carbon allocation between growth, storage carbohydrates, membrane lipids, neutral lipids, and high-value products such as EPA and fucoxanthin.
*Chlamydomonas*/*C. reinhardtii*	Nitrogen starvation, light-driven metabolism, trophic transitions, TAG/starch accumulation, and photosynthetic energy allocation	Genome-scale metabolic models, transcriptomics-constrained flux analysis, spectral light modeling, mutant phenotyping, targeted proteomics, metabolomics, and metabolic flux analysis	iRC1080 introduced wavelength-resolved photon constraints, allowing light quality to be translated into photon-utilizing metabolic reactions. iCre1355 integrated transcriptomic datasets to predict nitrogen-starvation responses and light-intensity-dependent pathway regulation. Nitrogen depletion suppresses growth-associated metabolism and chlorophyll biosynthesis while activating TAG/starch storage programs. Acyltransferase-associated regulation contributes to nitrogen-starvation-induced TAG accumulation. Proteomics/metabolomics/flux studies further support compartment-level regulation across chloroplast, mitochondrion, and cytosol.	*Chlamydomonas* provides the clearest example of integrating transcriptional regulation with constraint-based modeling to predict growth arrest, TAG accumulation, and light-dependent metabolic reprogramming. It also demonstrates that algal GEMs require explicit treatment of photon quality, energetic maintenance, and compartment-specific enzyme activity.
*Chlorella*/*C. vulgaris*, *C. variabilis*, *C. ohadii*	Trophic-mode flexibility, nitrogen starvation, medium optimization, light-quality effects, and extreme growth phenotypes	Genome-scale metabolic modeling, condition-specific biomass equations, RNA-seq-informed constraints, growth validation under trophic regimes, light-source simulations, enzyme-constrained modeling, and comparative flux analysis	In C. vulgaris, trophic mode changes flux through central carbon metabolism, amino acid metabolism, nucleotide biosynthesis, pigment metabolism, and lipid metabolism. Model-guided medium alteration identified tryptophan and methionine as growth-improving supplements. In C. variabilis, light-quality simulations show that red and blue wavelengths are important determinants of growth. In C. ohadii, enzyme-constrained modeling incorporates predicted catalytic rates and protein allocation, enabling comparison of growth strategies under standard and extreme light.	*Chlorella* species illustrate that regulatory behavior is strongly conditioned by trophic mode and light regime. Their models move from standard FBA toward condition-specific and enzyme-constrained frameworks, enabling prediction of medium effects, light-dependent growth differences, and potential targets for improving biomass productivity.
*Auxenochlorella*/*A. protothecoides*	Lutein production and model-guided identification of enzymatic regulators	Genome-scale metabolic modeling, multiple optimal-solution analysis, BRENDA-based enzyme-regulator screening, and experimental validation of medium additives	The iMM627 model was expanded to include lutein biosynthesis and used to identify reactions whose flux ranges differed between low- and high-lutein production states. Candidate reactions mapped to lutein biosynthesis, glycolysis, glyoxylate shunt, pentose phosphate pathway, purine metabolism, glycine metabolism, serine/threonine metabolism, and glutathione metabolism. Enzymatic activators were then selected using BRENDA and experimentally tested.	This work provides a direct example of translating flux-based predictions into enzyme-regulator interventions. Sodium citrate produced the strongest response, increasing lutein to 2.0868 mg/L and dry weight to 2.45 g/L, compared with 0.2233 mg/L lutein and 0.5 g/L dry weight in the control.
*Picochlorum*/*P. renovo*	Halotolerance, thermotolerance, outdoor-like diel productivity, and stress-adaptive metabolism	Genome analysis, physiological characterization, transformation tools, comparative transcriptomics under salinity stress, and biomass composition analysis	High-salinity responses include transcriptional changes in osmolyte-associated metabolism, especially proline-related pathways, together with candidate genes involved in membrane/sphingolipid remodeling, polyketide-associated secondary metabolism, DNA repair, and recombination. Physiological profiling shows growth under high salinity and high temperature, with dynamic allocation among carbohydrates, proteins, and lipids during diel cultivation.	Picochlorum expands the regulatory framework beyond nitrogen-starvation lipid accumulation. Its stress tolerance appears to rely on coordinated osmotic adjustment, membrane remodeling, secondary metabolism, and genome-protection mechanisms that allow sustained productivity under harsh saline and thermal regimes.
*Isochrysis*/*I. galbana*	Fucoxanthin production under phytohormone supplementation	Experimental phytohormone screening, biomass and pigment measurements, morphology analysis, and machine-learning prediction	Fucoxanthin production is modulated by phytohormone identity and concentration. Methyl jasmonate at 0.2 mg/L was identified as an effective condition for enhancing fucoxanthin yield. Random Forest models captured nonlinear relationships among growth, biomass, morphology, hormone treatment, and pigment output, improving prediction when hormone descriptors were included.	This example is not flux-based, but it supports the data-driven modeling component of predictive algal systems biology. It shows that ML can identify condition–product relationships for high-value pigments when mechanistic flux models are not yet available.
*Haematococcus*/*H. pluvialis*	Astaxanthin accumulation under light and nutrient stress	Multifactorial experimental design, ANN modeling, biomass/astaxanthin/nitrate/phosphate prediction, and multi-objective optimization	Astaxanthin production is separated from maximal biomass growth. Biomass accumulation is favored by nutrient availability, whereas astaxanthin accumulation is favored by high light and nitrate depletion with sufficient phosphate. ANN modeling predicted biomass, astaxanthin, nitrate, and phosphate dynamics across cultivation time.	The optimized astaxanthin condition produced 28.76 mg/L astaxanthin, equivalent to 3.06% dry weight, with 1.60 mg/L/day productivity. This illustrates a growth–secondary metabolism trade-off that can be captured by process-level ML and later linked to carotenoid-pathway regulation.

## Data Availability

No new data were created or analyzed in this study. This review is based exclusively on previously published literature cited in the manuscript. Data sharing is not applicable to this article.
